# A new class of Lindley distribution: System reliability, simulation and applications

**DOI:** 10.1016/j.heliyon.2024.e38335

**Published:** 2024-09-26

**Authors:** Danish Qayoom, Aafaq A. Rather, Najwan Alsadat, Eslam Hussam, Ahmed M. Gemeay

**Affiliations:** a Symbiosis Statistical Institute, Symbiosis International (Deemed University), Pune, 411004, India; b Department of Quantitative Analysis, College of Business Administration, King Saud University, P.O. Box 71115, Riyadh, 11587, Saudi Arabia; c Department of Mathematics, Faculty of Science, Helwan University, Cairo, Egypt; d Department of Mathematics, Faculty of Science, Tanta University, Tanta, 31527, Egypt

**Keywords:** Lindley distribution, DUS transformation, System reliability, Entropy, Bonferroni and lorenz curves, Simulation

## Abstract

This paper presents a new probability distribution called the DUS Lindley distribution, created by applying the DUS transformation to the traditional Lindley distribution. The study provides an in-depth analysis of the distribution's statistical properties. These properties include a variety of statistical measures such as the probability density function, cumulative distribution function, failure rate, survival function, reverse hazard function, Mills ratio, mean residual life, mean past life, moments, conditional moments, characteristic function, order statistics, entropy measures, likelihood ratio test and Lorenz and Bonferroni curves. Parameter estimation is performed using several methods including weighted least squares, maximum likelihood estimation, Cramer-Von Mises estimation, least squares and Anderson-Darling estimation. The paper also explores the estimation of system reliability and evaluates the performance of maximum likelihood estimators through simulation studies across different sample sizes. Finally, the DUS Lindley distribution is applied to two real-world datasets, demonstrating a better fit than other well-known distributions.

## 1 Introduction

Real-life data analysis is pivotal in numerous applied fields such as reliability analysis, biomedical research, engineering, and management social sciences. Effectively interpreting real-world phenomena involves employing a diverse array of probability distribution models. Among these, the most widely utilized probability models are exponential, gamma, normal, Weibull, Lindley, lognormal and many more. However, recent advancements have given rise to new generalized models, with particular attention being drawn to extensions of the Lindley distribution. The Lindley distribution and its generalizations have gained prominence due to their ability to model lifetime data effectively in numerous cases. Researchers are actively exploring and developing novel extensions of the Lindley distribution to enhance its applicability in describing lifetime phenomena. “The Lindley distribution, introduced by Lindley in 1958 [1], is formulated as a blend of exponential and gamma distributions. Ghitany et al. [2] delved into its structural properties and emphasized its appropriateness for modelling real-life data. Subsequent advancements include the power Lindley distribution proposed by Ghitany et al. [3] as a generalization of original Lindley distribution. Many other researchers have also explored diverse extensions of the Lindley distribution. These expansions encompass various modifications to the Lindley distribution. Nadarajah et al. [4] introduced the exponentiated Lindley, while Shanker and Mishra [5] proposed the Quasi-Lindley. Ashour and Eltehiwy [6] extended the exponentiated power Lindley distribution. Warahena-Liyanage and Pararai [7] developed the exponentiated power Lindley, and Alkarni [8] presented the extended inverse Lindley distribution. Alkarni [9] further contributed with the exponentiated power Lindley. Zeghdoudi and Nedjar [10] introduced the pseudo Lindley, and Nedjar and Zeghdoudi [11] proposed the gamma Lindley. Sharma et al. [12] developed the generalized inverse Lindley, while Alizadeh et al. [13] introduced the odd log-logistic power Lindley for bimodal data analysis. Aryuyuen [14] presented a Topp-Leone generator of the exponentiated power Lindley and its applications. Bicer [15] discussed statistical inference for the geometric process with the power Lindley distribution. Hassan et al. [16] explored the alpha power transformed power Lindley distribution, and Rather and Ozel [17] discussed the weighted power Lindley with applications in lifetime data, showcasing greater flexibility than classical distributions. Abdelall [18] discussed the Lomax–Lindley distribution. Rather and Ozel [19] introduced a new length-biased power Lindley distribution, providing insights into its properties and applications. Singh et al. [20] discussed the linear combination of order statistics of exponentiated Nadarajah-Haghighi distribution. Ahmad et al. [21,22]), obtained Novel Sin-G class of distributions with an illustration of Lomax distribution. Recently, Ali et al. [23], discussed on a transmuted distribution which is based on log-logistic and Ailamujia hazard functions with applications to lifetime data.

In recent years, the development of new probability distributions has become crucial for effectively modeling complex datasets that traditional models fail to adequately represent. This paper introduces the DUS Lindley distribution, derived by applying the DUS transformation to the Lindley distribution, to address these challenges. The motivation behind this work is to enhance flexibility and accuracy in statistical modeling by providing a robust tool for analyzing diverse data structures. The study offers a thorough examination of the DUS Lindley distribution's statistical properties, such as its density and distribution functions, failure rates, moments, and entropy measures. Advanced parameter estimation techniques, including maximum likelihood and weighted least squares, are employed to assess the distribution's applicability. Simulation studies validate the performance of these estimators, while real-world data applications demonstrate that the DUS Lindley distribution offers a superior fit compared to other existing models, showcasing its potential for widespread use in statistical analysis and reliability assessment. This work aims to enhance data modeling and analysis by offering a new, versatile tool for statisticians and researchers.

In this paper, the investigated model is achieved by applying the DUS transformation initially” proposed by Kumar et al. [24]. This transformative technique not only enhances the distribution's precision but also ensures computational simplicity and interpretation. Gul et al. [25] obtained DUS Inverse weibull. Later, Gul [26] extended the approach to DUS Weibull and DUS Inverse Weibull Distributions. Tripathi et al. [27] explored the DUS exponential distribution, focusing on upper record values for inference. In Bayesian survival analysis, AbuJarad et al. [28] worked on generalized DUS-exponential models. Recent studies by Deepti and Chacko (2020) [29], Gauthami and Chacko [30], and Kavya and Manoharan [31] have further contributed to exploring DUS transformation in various probability distribution models. Karakaya et al. [32] obtained the DUS-Kumaraswamy distribution. Thomas and Chacko [33] obtained the exponentiated version of DUS transformation known as power generalized DUS transformation.

The structure of this manuscript unfolds as: Section 2 delineates the derivation and graphical presentation of the probability density and cumulative distribution function. Section 3 focuses on reliability analysis and subsequent subsections. Section 4 delves into the statistical moments. System reliability estimation, with a focus on series systems, parallel systems, and series-parallel systems, is detailed in Section 5, while Sections 6, 7 are dedicated to order statistics and entropy measures, respectively. Lorenz and Bonferroni curves are expounded upon in Section 8. The estimation of parameters is addressed in Section 9, and other sub sections. Section 10 elucidates the Likelihood Ratio Test, and in section 11, a comprehensive simulation has been studied. Section 12 employs real-life data to assess the model's performance, and finally, Section 13 encapsulates conclusions and discussions derived from the study.

## 2 DUS lindley distribution

The probability density function (PDF) of Lindley distribution is

(1) $$f\left(z\;;\;\xi \right)=\left(\frac{\xi^{2}}{1+\xi }\right)\;\left(1+z\right)\;e^{-\xi \;z}\;;z>0,\xi >0$$

And the corresponding cumulative distribution function (CDF) of Lindley distribution is

(2) $$F_{Z}\left(z\;;\;\xi \right)=1-\left(1+\frac{\xi \;z}{1+\xi }\right)\;e^{-\xi \;z}$$

Now, the DUS model's PDF and CDF based on the existing distribution are generated using the following transformations, respectively.

(3) $$g\left(\;z\;\right)=\frac{1}{e-1}f\left(z\right)\;e^{F\left(z\right)}$$

and

(4) $$G\;\left(z\right)=\frac{1}{e-1}\;\left(e^{F\left(z\right)\;}-1\right)$$

On substituting equation (1) and equation (2) in equation (3), we get the PDF of the DUS Lindley distribution, which is mathematically expressed as

(5) $$g\left(z;\xi \right)=\left(\frac{1}{e-1}\right)\left(\frac{\xi^{2}}{1+\xi }\right)\;\left(1+z\right)\;e^{-\xi \;z}\;\left(e^{\left(1-\left(1+\frac{\xi \;z}{1+\xi }\right)\;e^{-\xi \;z}\right)}\right)$$

Similarly, on substituting equation (2) in equation (4) we get the CDF of the DUS Lindley distribution, which is given by

(6) $$G_{Z}\left(z\;;\;\xi \right)=\left(\frac{1}{e-1}\right)\;\left(e^{\left(1-\left(1+\frac{\xi \;z}{1+\xi }\right)\;e^{-\xi \;z}\right)}-1\right)$$

**Fig. 1:**
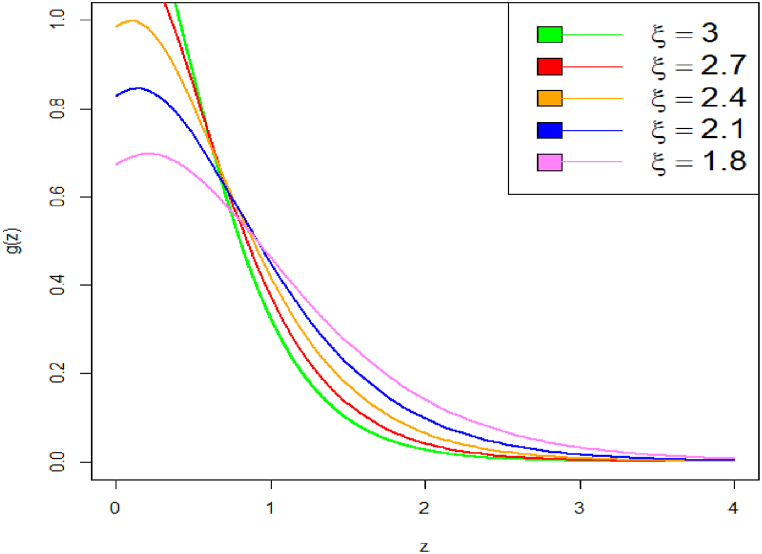
PDF plot of DUS Lindley distribution.

**Fig. 2:**
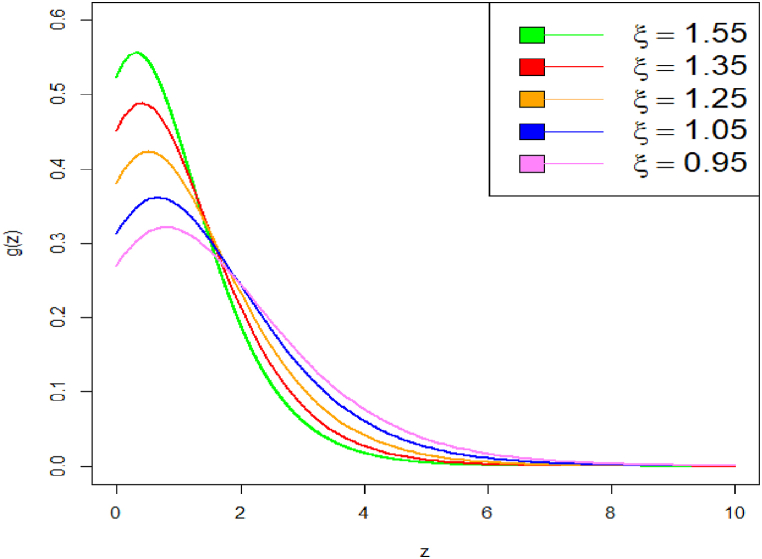
PDF plot of DUS Lindley distribution.

Graphical representation of PDF plot of DUS Lindley distribution is shown in Fig. 1, Fig. 2.

**Fig. 3:**
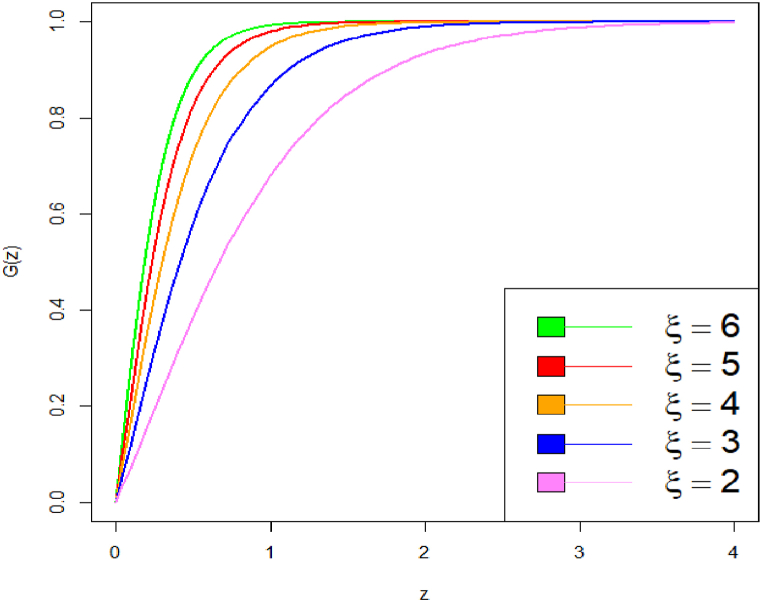
CDF plot of DUS Lindley distribution.

**Fig. 4:**
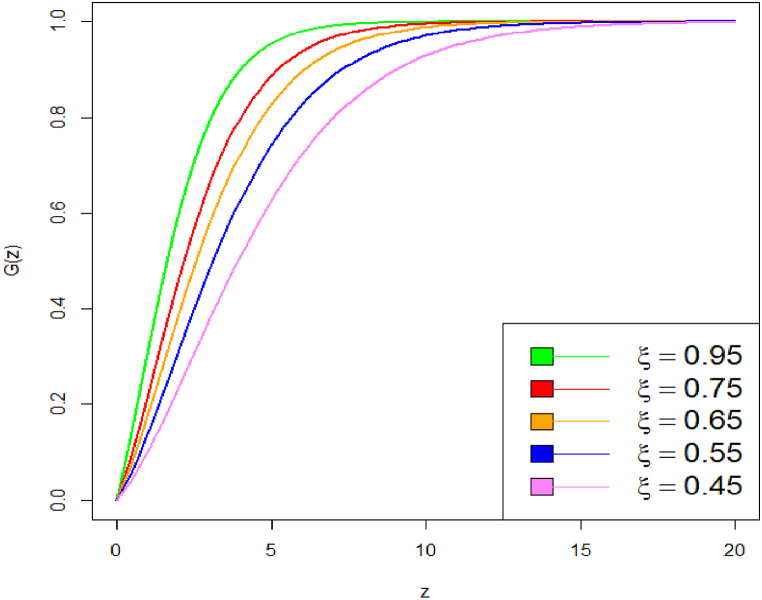
CDF plot of DUS Lindley distribution.

These plots highlight how changes in parameter values affect the distribution's shape and tail behavior. On decrease the value of parameter from say 3 to 1.8, the curve becomes heavy tailed towards right. Specifically, we have shown how decreasing the parameter values causes the PDF curve to become more right-tailed, indicating a shift in skewness. The corresponding CDF plot is shown in Fig. 3, Fig. 4.

## 3 Reliability analysis

Reliability analysis is the study and assessment of the reliability of systems, components, or processes to ensure their dependability and functionality over time. The main focus of reliability analysis is to identify potential failures, understand their causes, and implement measures to enhance the overall reliability of a system. Reliability analysis is essential in various industries, such as aerospace, automotive, electronics, telecommunications and healthcare, where system failures can have significant consequences. The various key aspects of reliability analysis are as.

### 3.1 Survival function

The survival function is a fundamental concept that describes the probability that a component or system will survive beyond a specific time. The survival function provides valuable information about the distribution of survival times within a population. The survival function of the DUS Lindley distribution is given by

$$S_{Z}\left(z\right)=1-G_{Z}\left(z\;;\;\xi \right)$$

On substituting equation (6) in the above equation we get

$$S_{Z}\left(z\right)=1-\left(\frac{1}{e-1}\right)\;\left(e^{\left(1-\left(1+\frac{\xi \;z}{1+\xi }\right)\;e^{-\xi \;z}\right)}-1\right)$$

**Fig. 5:**
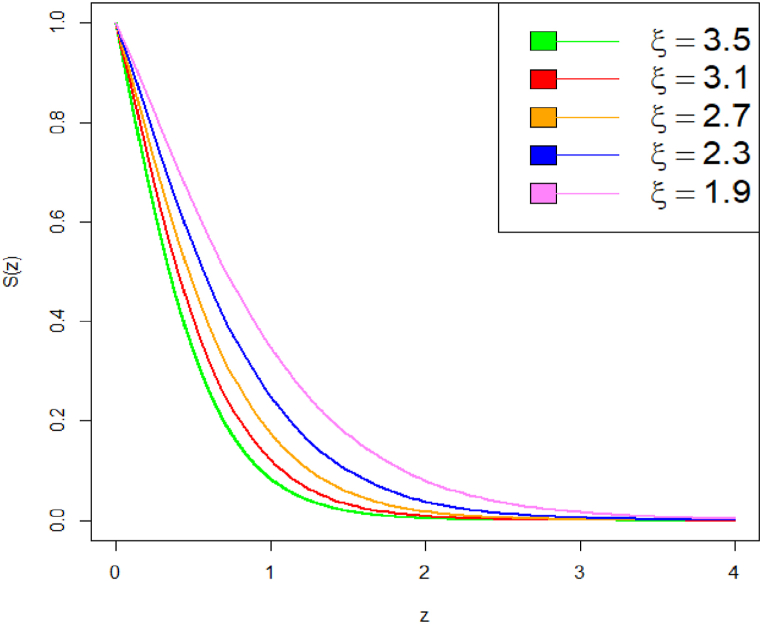
Survival function plot of DUS Lindley distribution.

**Fig. 6:**
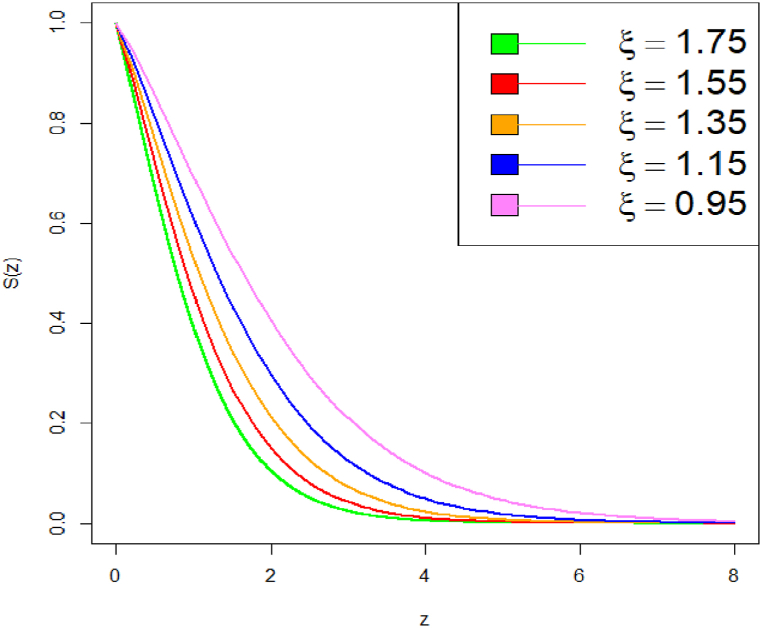
Survival function plot of DUS Lindley distribution.

Graphical representation of the survival function plot of DUS Lindley distribution is shown in Fig. 5, Fig. 6.

Comparing survival functions for different values of parameter can help in understanding how sensitive the survival probability is to these parameter values. It can be observed that a higher parameter value results in a more rapid decline of the survival function. The survival probability decreases more quickly, indicating that the event is expected to occur sooner. This suggests a higher hazard rate, meaning the event becomes more likely as time progresses. With a lower parameter value, the survival function declines more slowly, indicating a longer expected time until the event occurs. The hazard rate is lower, suggesting that the event is less likely to occur quickly and there is a longer period of survival.

### 3.2 Failure rate

The failure rate represents “the probability of failure per unit of time at a specific time point, given that the system has survived up to that point. The failure rate of the DUS Lindley distribution is given by

$$H_{Z}\left(z\right)=\frac{g\left(z\;;\;\xi \right)}{1-G_{Z}\left(z\;;\;\xi \right)}$$

Using equations (5), (6) in the above expression, we get

$$H_{Z}\left(z\right)=\frac{\left(\frac{1}{e-1}\right)\left(\frac{\xi^{2}}{1+\xi }\right)\;\left(1+z\right)\;e^{-\xi \;z}\;\left(e^{\left(1-\left(1+\frac{\xi \;z}{1+\xi }\right)\;e^{-\xi \;z}\right)}\right)}{1-\left(\frac{1}{e-1}\right)\;\left(e^{\left(1-\left(1+\frac{\xi \;z}{1+\xi }\right)\;e^{-\xi \;z}\right)}-1\right)}$$

After simplification, we get

$$H_{Z}\left(z\right)=\frac{\xi^{2}\left(1+z\right)\;e^{-\xi \;z}\left(e^{\left(1-\left(1+\frac{\xi \;z}{1+\xi }\right)\;e^{-\xi \;z}\right)}\right)}{\left(1+\xi \right)\;\left(e-\left(e^{\left(1-\left(1+\frac{\xi \;z}{1+\xi }\right)\;e^{-\xi \;z}\right)}\right)\right)}$$

### 3.3 Reverse hazard function

The reverse hazard function of the DUS Lindley distribution is

$$H_{Z}^{r}\left(z\right)=\frac{g\left(z\;;\;\xi \right)}{G_{Z}\left(z\right)}$$

$$H_{Z}^{r}\left(z\right)=\frac{\xi^{2}\left(1+z\right)\;e^{-\xi \;z}\left(e^{\left(1-\left(1+\frac{\xi \;z}{1+\xi }\right)\;e^{-\xi \;z}\right)}\right)}{\left(1+\xi \right)\;\left(e^{\left(1-\left(1+\frac{\xi \;z}{1+\xi }\right)\;e^{-\xi \;z}\right)}-1\right)}$$

### 3.4 Mills ratio

The mills ratio of DUS Lindley distribution is given by

$$Mills\;ratio=\frac{1}{H_{Z}^{r}\left(z\right)}$$

$$Mills\;ratio=\frac{\left(1+\xi \right)\;\left(e^{\left(1-\left(1+\frac{\xi \;z}{1+\xi }\right)\;e^{-\xi \;z}\right)}-1\right)}{\xi^{2}\left(1+z\right)\;e^{-\xi \;z}\left(e^{\left(1-\left(1+\frac{\xi \;z}{1+\xi }\right)\;e^{-\xi \;z}\right)}\right)}$$

### 3.5 Mean residual life

The Mean residual life (MRL) measures the expected remaining lifetime of a system or component, given that it has survived up to a certain point in time and helps in planning maintenance and replacement strategies. The MRL is used in various fields, such as actuarial research, forensic science, and reliability theory. For the DUS Lindley distribution, the MRL function can be derived as

MRL(t,ξ)=E(T−t|T>t)

$$MRL\left(t,\xi \right)=\frac{1}{1-G_{T}\left(t\right)}\int_{t}^{\infty }w\;g\left(w;\xi \right)dw-t$$

$$MRL\left(t,\xi \right)=\frac{1}{1-G_{T}\left(t\right)}\int_{t}^{\infty }w\left(\frac{1}{e-1}\right)\left(\frac{\xi^{2}}{1+\xi }\right)\;\left(1+w\right)\;e^{-\xi \;w}\;\left(e^{\left(1-\left(1+\frac{\xi \;w}{1+\xi }\right)\;e^{-\xi \;w}\right)}\right)dw-t$$

After simplification, we get

MRL(t,ξ)=11−GT(t)∑l=0∞∑m=0l∑k=0m(−1)l(Cml)(Ckm)(l!)(e−1)ξ(1+ξ)k+1((m+1)ξΓ(k+2,(m+1)ξt)+Γ(k+3,(m+1)ξt)(m+1)k+3)−t

Where Γ(k+2,(m+1)ξt) and Γ(k+3,(m+1)ξt) are upper incomplete gamma functions and are respectively given by

$$\Gamma \left(k+2,\left(m+1\right)\;\xi \;t\right)=\int_{\left(m+1\right)\;\xi \;t}^{\infty }w^{\left(k+2\right)-1}e^{-w}dw\;\text{and}\;\Gamma \left(k+3,\left(m+1\right)\;\xi \;t\right)=\int_{\left(m+1\right)\;\xi \;t}^{\infty }w^{\left(k+3\right)-1}e^{-w}dw$$

### 3.6 Mean past life

The expression of the mean past life (MPL) function for the DUS Lindley distribution is calculated as follows.

MPL(t,ξ)=E(t−T|T≤t)

$$MPL\left(t,\xi \right)=t-\frac{1}{G_{T}\left(t\right)}\int_{0}^{t}w\;g\left(w;\xi \right)dw$$

$$MPL\left(t,\xi \right)=t-\frac{1}{G_{T}\left(t\right)}\int_{0}^{t}w\left(\frac{1}{e-1}\right)\left(\frac{\xi^{2}}{1+\xi }\right)\;\left(1+w\right)\;e^{-\xi \;w}\;\left(e^{\left(1-\left(1+\frac{\xi \;w}{1+\xi }\right)\;e^{-\xi \;w}\right)}\right)dw$$

After simplification, we get.

MPL(t,ξ)=t−1GT(t)∑l=0∞∑m=0l∑k=0m(−1)l(Cml)(Ckm)(l!)(e−1)ξ(1+ξ)k+1((m+1)ξγ(k+2,(m+1)ξt)+γ(k+3,(m+1)ξt)(m+1)k+3) Where γ(k+2,(m+1)ξt) and γ(k+3,(m+1)ξt) are lower incomplete gamma functions and are respectively given by

$$\gamma \left(k+2,\left(m+1\right)\;\xi \;t\right)=\int_{0}^{\left(m+1\right)\;\xi \;t}w^{\left(k+2\right)-1}e^{-w}dw\;\text{and}\;\gamma \left(k+3,\left(m+1\right)\;\xi \;t\right)=\int_{0}^{\left(m+1\right)\;\xi \;t}w^{\left(k+3\right)-1}e^{-w}dw$$

## 4 Statistical properties

### 4.1 Moments

The *rth* raw moment about the origin of DUS Lindley distribution is

$$\mu_{r}^{\prime }=\int_{0}^{\infty }z^{r}\left(\frac{1}{e-1}\right)\left(\frac{\xi^{2}}{1+\xi }\right)\;\left(1+z\right)\;e^{-\xi \;z}\;\left(e^{\left(1-\left(1+\frac{\xi \;z}{1+\xi }\right)\;e^{-\xi \;z}\right)}\right)dz$$

After simplification, we get

(7) μr′=∑l=0∞∑m=0l∑k=0m(−1)l(Cml)(Ckm)Γ(k+r+1)(l!)(e−1)ξr(1+ξ)k+1((m+1)ξ+(k+r+1)(m+1)k+r+2)

Substituting r=1,2,3,4 in equation (7), the first four moments about the origin of the given distribution and are summarised below.

μ1′=∑l=0∞∑m=0l∑k=0m(−1)l(Cml)(Ckm)Γ(k+2)(l!)(e−1)ξ(1+ξ)k+1((m+1)ξ+k+2(m+1)k+3)

μ2′=∑l=0∞∑m=0l∑k=0m(−1)l(Cml)(Ckm)Γ(k+3)(l!)(e−1)ξ2(1+ξ)k+1((m+1)ξ+k+3(m+1)k+4)

μ3′=∑l=0∞∑m=0l∑k=0m(−1)l(Cml)(Ckm)Γ(k+4)(l!)(e−1)ξ3(1+ξ)k+1((m+1)ξ+k+4(m+1)k+5)

μ4′=∑l=0∞∑m=0l∑k=0m(−1)l(Cml)(Ckm)Γ(k+5)(l!)(e−1)ξ4(1+ξ)k+1((m+1)ξ+k+5(m+1)k+6)

### 4.2 Conditional moments

The rth conditional moment with parameter ξ of DUS Lindley distribution can be calculated as

$$E\left(Z^{r}|Z>z\right)=\frac{1}{1-G_{Z}\left(z\right)}\int_{z}^{\infty }w^{r}g\left(w\;;\;\xi \right)dw$$

$$E\left(Z^{r}|Z>z\right)=\frac{1}{1-G_{Z}\left(z\right)}\int_{z}^{\infty }w^{r}\left(\frac{1}{e-1}\right)\left(\frac{\xi^{2}}{1+\xi }\right)\;\left(1+w\right)\;e^{-\xi \;w}\;\left(e^{\left(1-\left(1+\frac{\xi \;w}{1+\xi }\right)\;e^{-\xi \;w}\right)}\right)dw$$

After simplification, we get

E(Zr|Z>z)=(11−GZ(z))∑l=0∞∑m=0l∑k=0m(−1)l(Cml)(Ckm)(l!)(e−1)(ξ)r(1+ξ)k+1((m+1)ξzΓ(k+r+1,(m+1)ξz)+Γ(k+r+2,(m+1)ξz)(m+1)k+r+2)

Where Γ(k+r+1,(m+1)ξz) and Γ(k+r+2,(m+1)ξz) are upper incomplete gamma functions and are respectively given by

$$\Gamma \left(k+r+1,\left(m+1\right)\;\xi \;z\right)=\int_{\left(m+1\right)\;\xi \;z}^{\infty }t^{\left(k+r+1\right)-1}e^{-t}dt\;\text{and}\;\Gamma \left(k+r+2,\left(m+1\right)\;\xi \;z\right)=\int_{\left(m+1\right)\;\xi \;z}^{\infty }t^{\left(k+r+2\right)-1}e^{-t}dt$$

The Moment generating function (m.g.f) of the DUS Lindley distribution” is

$$M_{Z}\left(t\right)=\int_{0}^{\infty }e^{t\;z}\left(\frac{1}{e-1}\right)\left(\frac{\xi^{2}}{1+\xi }\right)\;\left(1+z\right)\;e^{-\xi \;z}\;\left(e^{\left(1-\left(1+\frac{\xi \;z}{1+\xi }\right)\;e^{-\xi \;z}\right)}\right)\;dz$$

After simplification

MZ(t)=∑r=o∞∑l=0∞∑m=0l∑k=0m(tr)(−1)l(Cml)(Ckm)Γ(k+r+1)(r!)(l!)(e−1)ξr(1+ξ)k+1((m+1)ξ+(k+r+1)(m+1)k+r+2)

The characteristics function of the DUS Lindley distribution is

$$\varphi_{Z}\left(t\right)=\int_{0}^{\infty }e^{\iota \;t\;z}\left(\frac{1}{e-1}\right)\left(\frac{\xi^{2}}{1+\xi }\right)\;\left(1+z\right)\;e^{-\xi \;z}\;\left(e^{\left(1-\left(1+\frac{\xi \;z}{1+\xi }\right)\;e^{-\xi \;z}\right)}\right)\;dz$$

After simplification

φZ(t)=∑r=o∞∑l=0∞∑m=0l∑k=0m(ιt)r(−1)l(Cml)(Ckm)Γ(k+r+1)(r!)(l!)(e−1)ξr(1+ξ)k+1((m+1)ξ+(k+r+1)(m+1)k+r+2)

The cumulant generating function of DUS Lindley distribution is

$$\kappa_{Z}\left(t\right)=\log\left(M_{Z}\left(t\right)\right)$$

κZ(t)=log(∑r=o∞∑l=0∞∑m=0l∑k=0m(tr)(−1)l(Cml)(Ckm)Γ(k+r+1)(r!)(l!)(e−1)ξr(1+ξ)k+1((m+1)ξ+(k+r+1)(m+1)k+r+2))

## 5 System reliability estimation

System reliability shows the ability of a system to consistently perform its intended functions without failure over a specified time. It is a paramount consideration in designing, implementing, and maintaining various infrastructures across diverse industries. Whether it is an aerospace system, a power grid, a data centre, or a manufacturing facility, the reliability of a system directly impacts its performance, safety, and overall success. Achieving and maintaining high reliability involves strategic planning, system structure, rigorous testing, proactive maintenance, and continuous improvement. In this section, we will explore system reliability according to distinct structures. Many researchers have contributed a lot in this field, including Ghare and Taylor [34], Banerjee and Rajamani (1973), Bai et al. [35], Petrovic [36], Kuo and Prasad [37], Utkin and Gurov [38], Lee et al.[39], Hoang Pham[40], Bhunia et al. [41], Bakshi et al. (2011), Ahmad et al. [21,22]).

### 5.1 Series system

**Fig. 7:**
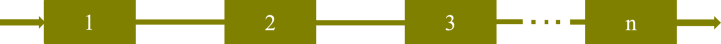
Series system structure.

In reliability analysis, a series system is characterized by a sequential arrangement of components. The system is deemed operational as long as each component functions smoothly. However, a breakdown in any single component results in the system's overall failure; hence, the strength of the entire system is determined by its weakest component. A series system structure is shown in Fig. 7 below:

The reliability of a system with n components connected in series is the product of the individual component reliabilities. That is,

$$R_{s}\left(t\right)=r_{1}\left(t\right)\times r_{2}\left(t\right)\times r_{3}\left(t\right)\times ...\times r_{n}\left(t\right)$$

(8) $$R_{s}\left(t\right)=\prod_{j=1}^{n}r_{j}\left(t\right)$$

Let us assume the system components are identically independent and follows the DUS Lindley distribution. Then, for each component the reliability will be

(9) $$r_{j}\left(t\right)=1-\left(\frac{1}{e-1}\right)\left(e^{\left(1-\left(1+\frac{{\xi t}_{j}}{1+\xi }\right)e^{{-\xi t}_{j}}\right)}-1\right);j=1,2,3,\cdots ,n$$

On substituting equation (9) in equation (8) we get

(10) $$R_{s}\left(t\right)=\Pi_{j=1}^{n}\left(1-\left(\frac{1}{e-1}\right)\left(e^{\left(1-\left(1+\frac{{\xi t}_{j}}{1+\xi }\right)e^{{-\xi t}_{j}}\right)}-1\right)\right)$$

The expression in equation (10) represents the reliability function associated with the system whose components are connected in series.

### 5.2 Parallel system

**Fig. 8:**
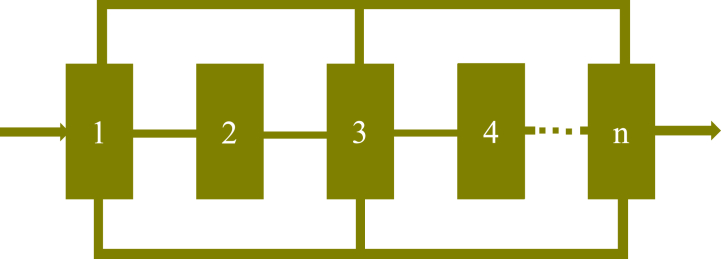
Parallel system structure.

Parallel systems play a crucial role in reliability analysis, particularly in designing systems that can continue functioning even if individual components fail. In a parallel system, the components or subsystems operate independently, and the system functions as long as at least one is operational. This system design enhances fault tolerance and availability and introduces a streamlined approach to maintenance, ensuring a resilient and user-friendly system. A parallel system design is shown in Fig. 8 below:

In the case of a parallel system with n components, the reliability will be

$$R_{s}\left(t\right)=1-\left(1-R_{1}\left(t\right)\right)\;\left(1-R_{2}\left(t\right)\right)\;\left(1-R_{3}\left(t\right)\right)\;...\left(1-R_{n}\left(t\right)\right)$$

(11) $$R_{s}\left(t\right)=1-\prod_{j=1}^{n}\left(1-R_{j}\left(t\right)\right)$$

Suppose the system components are independent and are identically distributed with DUS Lindley distribution. Then, using equation (9) in equation (11), the reliability will be

(12) $$R_{s}\left(t\right)=1-\Pi_{j=1}^{n}\left(1-\left(1-\left(\frac{1}{e-1}\right)\left(e^{\left(1-\left(1+\frac{{\xi t}_{j}}{1+\xi }\right)e^{{-\xi t}_{j}}\right)}-1\right)\right)\right)$$

So, equation (12) represents the reliability function of the system whose components are connected in parallel and follow DUS Lindley distribution.

### 5.3 Series-parallel system

**Fig. 9:**
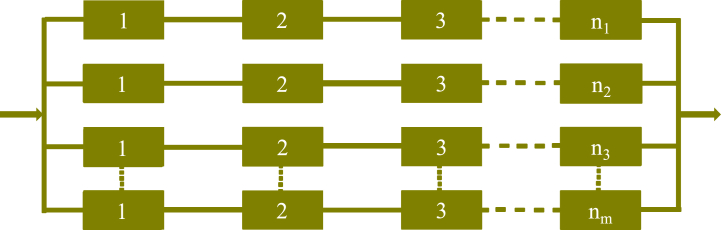
Series-Parallel system structure.

A series-parallel system combines both series and parallel configurations to optimize performance. The primary purpose of using this hybrid arrangement is to enhance reliability, efficiency, and functionality. Understanding the specific application of series-parallel systems can provide more insights into their design and implementation. Common applications of series-parallel systems include electrical power distribution networks, automotive electrical systems, computer networks, industrial machinery, telecommunications and many more. In a series-parallel arrangement, the components or subsystems are first connected in series, and then these series configurations are linked together in parallel, as shown in Fig. 9 below:

Let us consider *m* subsystems connected in parallel and assume that each subsystem has *n*_*i*_ (*i* = *1, 2, …m*) components connected in series. This type of system is known as series-parallel system. Let *R*_*i*_*(t)* be the reliability of subsystem *i* and *r*_*ij*_*(t)* be the reliability of *j*^*th*^ component of *i*^*th*^ subsystem, *j=1,2, …n*_*i*_. Then

$$R_{i}\left(t\right)=\prod_{j=1}^{n_{i}}r_{i\;j}\left(t\right)$$

Therefore, the reliability of the series-parallel system is

$$R_{s}\left(t\right)=1-\prod_{i=1}^{m}\left(1-R_{i}\left(t\right)\right)$$

(13) $$R_{s}\left(t\right)=1-\prod_{i=1}^{m}\left(1-\prod_{j=1}^{n_{i}}r_{i\;j}\left(t\right)\right)$$

Suppose the system components are identically independent and follows the DUS Lindley distribution. Then, the reliability of each component will be

(14) $$r_{ij}\left(t\right)=1-\left(\frac{1}{e-1}\right)\left(e^{\left(1-\left(1+\frac{{\xi t}_{j}}{1+\xi }\right)e^{{-\xi t}_{j}}\right)}-1\right)$$

Substituting equation (14) in equation (13), we get.

$R_{s}\left(t\right)=1-\prod_{i=1}^{m}\left(1-\prod_{j=1}^{n_{i}}\left(1-\left(\frac{1}{e-1}\right)\;\left(e^{\left(1-\left(1+\frac{\xi \;t_{j}}{1+\xi }\right)\;e^{-\xi \;t_{j}}\right)}-1\right)\right)\right)$; if all components in subsystem i are independent

or.

$R_{s}\left(t\right)=1-\prod_{i=1}^{m}\left(1-{\left(1-\left(\frac{1}{e-1}\right)\;\left(e^{\left(1-\left(1+\frac{\xi \;t_{j}}{1+\xi }\right)\;e^{-\xi \;t_{j}}\right)}-1\right)\right)}^{n_{i}}\right)\;$; if all components are i.i.d and subsystems are not the same.

Example 1 System Reliability

In this section, we will give different parameter values in the below reliability function and fix the value of t to understand how the system or component is functioning.

$$r\left(t\right)=1-\left(\frac{1}{e-1}\right)\left(e^{\left(1-\left(1+\frac{{\xi t}_{j}}{1+\xi }\right)e^{{-\xi t}_{j}}\right)}-1\right)$$

Let t=300,θ=0.001, we get

$$r_{1}\left(t\right)=1-\left(\frac{1}{e-1}\right)\;\left(e^{\left(1-\left(1+\frac{0.001\times 300}{1+0.001}\right)e^{-0.001\times 300\;}\right)}-1\right)$$

$$r_{1}\left(t\right)=1-\left(\frac{1}{2.718-1}\right)\;\left(e^{0.0372}-1\right)$$

$$r_{1}\left(t\right)=0.9779$$

For t=300,θ=0.002, we get

$$r_{2}\left(t\right)=0.9241$$

For t=300,θ=0.003, we get

$$r_{3}\left(t\right)=0.8505$$

Let t=400,θ=0.001, we get

$$r_{1}\left(t\right)=1-\left(\frac{1}{e-1}\right)\;\left(e^{\left(1-\left(1+\frac{0.001\times 400}{1+0.001}\right)e^{-0.001\times 400\;}\right)}-1\right)$$

$$r_{1}\left(t\right)=1-\left(\frac{1}{2.718-1}\right)\;\left(e^{0.0618}-1\right)$$

$$r_{1}\left(t\right)=0.9629$$

For t=400,θ=0.002, we get

$$r_{2}\left(t\right)=0.8769$$

For t=400,θ=0.003, we get

$$r_{3}\left(t\right)=0.7656$$

The values of r_1_(t)*,* r_2_(t) and r_3_(t) typically indicates that the probability of a system or component is functioning correctly.

## 6 Ordered statistics

Suppose “*z*_*1*_*, z*_*2*_*, z*_*3*_*, …, z*_*n*_ be the random sample drawn from the DUS Lindley distribution. Then Z_(1)_, Z_(2)_, Z_(3)_, …, Z_(n)_ be the ordered statistics associated with a random sample of size n selected from the given probability distribution such that.

Z_(1)_ = min(*z*_*1*_*, z*_*2*_*, z*_*3*_*, …, z*_*n*_) and Z_(n)_ = max(*z*_*1*_*, z*_*2*_*, z*_*3*_*, …, z*_*n*_)

The PDF of the *r*^*th*^-ordered statistics is given by

$$g_{Z_{\left(r\right)}}\left(z\right)=\frac{n!}{\left(n-r\right)!\left(r-1\right)!}g\left(z\;;\;\xi \right){\left(G_{Z}\left(z\;;\;\xi \right)\right)}^{r-1}{\left(1-G_{Z}\left(z\;;\;\xi \right)\right)}^{n-r}$$

(15) $$g_{Z_{\left(r\right)}}\left(z\right)=\frac{n!}{\left(n-r\right)!\left(r-1\right)!}\left(\frac{1}{e-1}\right)\left(\frac{\xi^{2}}{1+\xi }\right)\;\left(1+z\right)\;e^{-\xi \;z}\;\left(e^{\left(1-\left(1+\frac{\xi \;z}{1+\xi }\right)\;e^{-\xi \;z}\right)}\right)\times {\left(\left(\frac{1}{e-1}\right)\;\left(e^{\left(1-\left(1+\frac{\xi \;z}{1+\xi }\right)\;e^{-\xi \;z}\right)}-1\right)\right)}^{r-1}{\left(1-\left(\frac{1}{e-1}\right)\;\left(e^{\left(1-\left(1+\frac{\xi \;z}{1+\xi }\right)\;e^{-\xi \;z}\right)}-1\right)\right)}^{n-r}$$

The corresponding CDF of *r*th-ordered statistics is

GZ(r)(z)=∑j=rn(Crn)(GZ(z;ξ))j(1−GZ(z;ξ))n−j

GZ(r)(z)=∑j=rn(Crn)((1e−1)(e(1−(1+ξz1+ξ)e−ξz)−1))j(1−(1e−1)(e(1−(1+ξz1+ξ)e−ξz)−1))n−j

Put *r = n* in equation (15), we obtain the highest ordered statistics PDF of DUS Lindley distribution and is equal to

$$g_{Z_{\left(n\right)}}\left(z\right)=n\left(\frac{1}{e-1}\right)\left(\frac{\xi^{2}}{1+\xi }\right)\;\left(1+z\right)\;e^{-\xi \;z}\;\left(e^{\left(1-\left(1+\frac{\xi \;z}{1+\xi }\right)\;e^{-\xi \;z}\right)}\right)\;{\left(\left(\frac{1}{e-1}\right)\;\left(e^{\left(1-\left(1+\frac{\xi \;z}{1+\xi }\right)\;e^{-\xi \;z}\right)}-1\right)\right)}^{n-1}$$

And the corresponding CDF of highest ordered statistics is

$$G_{Z_{\left(n\right)}}\left(z\right)={\left(\left(\frac{1}{e-1}\right)\;\left(e^{\left(1-\left(1+\frac{\xi \;z}{1+\xi }\right)\;e^{-\xi \;z}\right)}-1\right)\right)}^{n}$$

Also, the PDF of smallest ordered statistics *Z*_(*1*)_ of DUS Lindley distribution is

$$g_{Z_{\left(1\right)}}\left(z\right)=n\left(\frac{1}{e-1}\right)\left(\frac{\xi^{2}}{1+\xi }\right)\;\left(1+z\right)\;e^{-\xi \;z}\;\left(e^{\left(1-\left(1+\frac{\xi \;z}{1+\xi }\right)\;e^{-\xi \;z}\right)}\right){\left(1-\left(\frac{1}{e-1}\right)\;\left(e^{\left(1-\left(1+\frac{\xi \;z}{1+\xi }\right)\;e^{-\xi \;z}\right)}-1\right)\right)}^{n-1}$$

And the corresponding CDF of smallest ordered statistics” *Z*_(*1*)_ is

$$G_{Z_{\left(r\right)}}\left(z\right)=1-{\left(1-\left(\frac{1}{e-1}\right)\;\left(e^{\left(1-\left(1+\frac{\xi \;z}{1+\xi }\right)\;e^{-\xi \;z}\right)}-1\right)\right)}^{n}$$

## 7 Entropy measures

Entropy measures are quantitative metrics used to describe the uncertainty or randomness of a probability distribution or dataset. In information theory, entropy quantifies the average amount of information produced by a stochastic source of data, reflecting the unpredictability of the data's outcomes. Higher entropy indicates greater disorder and uncertainty, while lower entropy suggests more predictability and order. In statistics and data analysis, entropy measures are often used to assess the variability or diversity of a dataset, helping to identify patterns or the level of complexity in the data.

### 7.1 Renyi entropy

The Renyi entropy of the DUS Lindley distribution is

$$\Lambda \left(\varsigma \right)=\frac{1}{1-\varsigma }\log\left(\int_{0}^{\infty }{\left(g\left(z\;;\;\xi \right)\right)}^{\varsigma }dz\right)$$

$$\Lambda \left(\varsigma \right)=\frac{1}{1-\varsigma }\log\left(\int_{0}^{\infty }{\left(\left(\frac{1}{e-1}\right)\left(\frac{\xi^{2}}{1+\xi }\right)\;\left(1+z\right)\;e^{-\xi \;z}\;\left(e^{\left(1-\left(1+\frac{\xi \;z}{1+\xi }\right)\;e^{-\xi \;z}\right)}\right)\right)}^{\varsigma }dz\right)$$

After simplification, we get

Λ(ς)=11−ςlog(∑l=0∞∑m=0l∑k=0m∑j=0∞(−ς)l(Cml)(Ckm)(Cjς)(ξ)2ς−j−1Γ(k+j+1)(l!)(e−1)ς(1+ξ)ς+k(m+ς)k+j+1)

### 7.2 Tsallis entropy

The Tsallis entropy of the DUS Lindley distribution is

$$\Delta \left(\vartheta \right)=\frac{1}{\vartheta -1}\left(1-\int_{0}^{\infty }{\left(g\left(z\;;\;\xi \right)\right)}^{\vartheta }dz\right)$$

$$\Delta \left(\vartheta \right)=\frac{1}{\vartheta -1}\left(1-\int_{0}^{\infty }{\left(\left(\frac{1}{e-1}\right)\left(\frac{\xi^{2}}{1+\xi }\right)\;\left(1+z\right)\;e^{-\xi \;z}\;\left(e^{\left(1-\left(1+\frac{\xi \;z}{1+\xi }\right)\;e^{-\xi \;z}\right)}\right)\right)}^{\vartheta }dz\right)$$

After simplification, we get

Δ(ϑ)=1ϑ−1(1−∑l=0∞∑m=0l∑k=0m∑j=0∞(−ϑ)l(Cml)(Ckm)(Cjϑ)(ξ)2ϑ−j−1Γ(k+j+1)(l!)(e−1)ϑ(1+ξ)ϑ+k(m+ϑ)k+j+1)

## 8 Lorenz and Bonferroni curves

The Lorenz curve is a graphical representation used to illustrate income or wealth distribution within a population, plotting the cumulative percentage of total income or wealth against the cumulative percentage of the population. It helps visualize inequality, where a perfectly equal distribution would result in a 45-degree line.

The Bonferroni curve is a variation of the Lorenz curve that highlights extreme inequalities, particularly in the lower tail of the distribution. It is used to assess the concentration of poverty or inequality among the poorest segments of a population.

### 8.1 Lorenz curve

The Lorenz curve associated with the DUS Lindley distribution is given by

$$L_{c}\left(\eta \right)=\frac{1}{\mu_{1}^{\prime }}\left(\int_{0}^{\Psi }z\;g\left(z\;;\;\xi \right)dz\right)$$

Where

μ1′=∑l=0∞∑m=0l∑k=0m(−1)l(Cml)(Ckm)Γ(k+2)(l!)(e−1)ξ(1+ξ)k+1((m+1)ξ+k+2(m+1)k+3)andΨ=F−1(η)

$$L_{c}\left(\eta \right)=\frac{1}{\mu_{1}^{\prime }}\left(\int_{0}^{\Psi }z\;\left(\frac{1}{e-1}\right)\left(\frac{\xi^{2}}{1+\xi }\right)\;\left(1+z\right)\;e^{-\xi \;z}\;\left(e^{\left(1-\left(1+\frac{\xi \;z}{1+\xi }\right)\;e^{-\xi \;z}\right)}\right)dz\right)$$

After simplification, we get

Lc(η)=1μ1′(∑l=0∞∑m=0l∑k=0m(−1)l(Cml)(Ckm)(l!)(e−1)ξ(1+ξ)k+1((m+1)ξγ(k+2,(m+1)ξΨ)+γ(k+3,(m+1)ξψ)(m+1)k+3))

### 8.2 Bonferroni curve

The Bonferroni curve associated with the DUS Lindley distribution is given by

$$B_{c}\left(\eta \right)=\frac{1}{\eta }L_{c}\left(\eta \right)$$

Bc(η)=1ημ1′(∑l=0∞∑m=0l∑k=0m(−1)l(Cml)(Ckm)(l!)(e−1)ξ(1+ξ)k+1((m+1)ξγ(k+2,(m+1)ξΨ)+γ(k+3,(m+1)ξψ)(m+1)k+3))

## 9 Parameter estimation

### 9.1 Maximum likelihood estimation (MLE)

Maximum Likelihood Estimation (MLE) is a “versatile and widely applicable method crucial in statistical modelling and parameter estimation. The likelihood function of a random sample of size *n* from the DUS Lindley distribution is

$$L\left(\xi \right)=\prod_{i=1}^{n}\left(\frac{1}{e-1}\right)\left(\frac{\xi^{2}}{1+\xi }\right)\;\left(1+z_{i}\right)\;e^{-\xi \;z_{i}}\;\left(e^{\left(1-\left(1+\frac{\xi \;z_{i}}{1+\xi }\right)\;e^{-\xi \;z_{i}}\right)}\right)$$

$$L\left(\xi \right)={\left(\left(\frac{1}{e-1}\right)\left(\frac{\xi^{2}}{1+\xi }\right)\right)}^{n}\left(\prod_{i=1}^{n}\left(1+z_{i}\right)\right)\;\left(e^{-\xi \sum_{i=1}^{n}z_{i}\;}\right)\;\left(e^{\sum_{i=1}^{n}\left(1-\left(1+\frac{\xi \;z_{i}}{1+\xi }\right)e^{-\xi \;z_{i}}\right)}\right)$$

Applying log on both sides we get

(16) $$\log\;L\left(\xi \right)=n\;\log\left(\frac{1}{e-1}\right)+n\;\log\left(\frac{\xi^{2}}{1+\xi }\right)+\sum_{i=1}^{n}\log\left(1+z_{i}\right)-\xi \sum_{i=1}^{n}z_{i}+\sum_{i=1}^{n}\left(1-\left(1+\frac{\xi \;z_{i}}{1+\xi }\right)e^{-\xi \;z_{i}}\right)$$

Differentiating equation (16) partially w.r.t *ξ* and equating to zero, we get

$$\frac{2n}{\xi }-\frac{n}{1+\xi }-\sum_{i=1}^{n}z_{i}-\sum_{i=1}^{n}\left(\left(1+\frac{\xi \;z_{i}}{1+\xi }\right)\;e^{-\xi \;z_{i}}\left(-z_{i}\right)+e^{-\xi \;z_{i}}\left(\frac{z_{i}}{{\left(1+\xi \right)}^{2}}\right)\right)=0$$

$$\frac{2n}{\xi }-\frac{n}{1+\xi }-\sum_{i=1}^{n}z_{i}+\sum_{i=1}^{n}z_{i}\left(1+\frac{\xi \;z_{i}}{1+\xi }\right)\;e^{-\xi \;z_{i}}-\sum_{i=1}^{n}\left(\frac{z_{i}}{{\left(1+\xi \right)}^{2}}\right)e^{-\xi \;z_{i}}=0$$

Because of the complicated form of the above algebraic expression, we use statistical software such as R, Mathematica and Newton-Raphson methods for estimating the given parameter.

### 9.2 Least squares estimation (LSE)

Sppose Z_(1)_, Z_(2)_, Z_(3)_, …, Z_(n)_ be the ordered statistics associated with a random sample of size n selected from probability distribution. Then, the ordinary least square estimator of the unknown parameter involved in the distribution is obtained by minimizing the error sum of squares.

$E=\sum_{r=1}^{n}{\left(G_{Z_{\left(r\right)}}\left(z\right)-E\left(G_{Z_{\left(r\right)}}\left(z\right)\right)\right)}^{2}$ with respect to that unknown parameter.Where $E\left(G_{Z_{\left(r\right)}}\left(z\right)\right)=\frac{r}{n+1}$ and $G_{Z_{\left(r\right)}}\left(z\right)$ is the CDF of rth ordered statistic. So, the ordinary least squares estimator of the unknown parameter ξ of the DUS Lindley distribution is obtained by minimizing

E(ξ|z_)=∑r=1n(∑j=rn(Crn)((1e−1)(e(1−(1+ξz1+ξ)e−ξz)−1))j(1−(1e−1)(e(1−(1+ξz1+ξ)e−ξz)−1))n−j−rn+1)2 with respect to parameter ξ.

### 9.3 Weighted least squares estimation (WLSE)

The weighted least squares estimator of the unknown parameter of the distribution is obtained by minimizing the.

$E_{W}=\sum_{r=1}^{n}\left(\frac{1}{V\left(G_{Z_{\left(r\right)}}\left(z\right)\right)}\right){\left(G_{Z_{\left(r\right)}}\left(z\right)-E\left(G_{Z_{\left(r\right)}}\left(z\right)\right)\right)}^{2}$ with respect to that unknown parameter.

Where $V\left(G_{Z_{\left(r\right)}}\left(z\right)\right)=\frac{r\left(n-r+1\right)}{{\left(n+1\right)}^{2}\left(n+2\right)}$. So, in the case of the DUS Lindley distribution, the weighted least squares estimator of the unknown parameter ξ is obtained by minimizing the function

EW(ξ|z_)=∑r=1n((n+1)2(n+2)r(n−r+1))(∑j=rn(Crn)((1e−1)(e(1−(1+ξz1+ξ)e−ξz)−1))j(1−(1e−1)(e(1−(1+ξz1+ξ)e−ξz)−1))n−j−rn+1)2 with respect to parameter ξ.

### 9.4 Cramer-von Mises Estimation (CVME)

The Cramér-von Mises Estimation (CVME) is a statistical method used to evaluate the goodness-of-fit of a distribution to a set of data by measuring the squared differences between the empirical cumulative distribution function (ECDF) and the theoretical cumulative distribution function (CDF) of the hypothesized model. Unlike the Kolmogorov-Smirnov test, CVME focuses on the overall discrepancies across the entire range of data, not just the maximum difference, making it sensitive to deviations throughout the distribution. It is widely used in goodness-of-fit testing to provide a more balanced assessment of fit quality.

The Cramer-Von Mises estimator of the unknown parameter of distribution is obtained as

$$\Phi_{CM}=\frac{1}{12n}+\sum_{r=1}^{n}{\left(G_{Z_{\left(r\right)}}\left(z\right)-\left(\frac{2r-1}{2n}\right)\right)}^{2}$$

Therefore, in the case of the DUS Lindley distribution, the Cramer-Von Mises estimator of unknown parameter ξ is obtained by minimizing.

ΦCM(ξ|z_)=112n+∑r=1n(∑j=rn(Crn)((1e−1)(e(1−(1+ξz1+ξ)e−ξz)−1))j(1−(1e−1)(e(1−(1+ξz1+ξ)e−ξz)−1))n−j−(2r−12n))2 with respect to ξ.

### 9.5 Anderson-Darling estimation (ADE) method

The Anderson-Darling Estimation (ADE) method is a statistical technique used to assess the goodness-of-fit of a distribution to a sample of data. It enhances the basic chi-square or Kolmogorov-Smirnov tests by giving more weight to the tails of the distribution. The ADE method measures the distance between the empirical cumulative distribution function (ECDF) and the theoretical CDF, providing a more sensitive detection of discrepancies, especially in the tails, which is crucial in reliability studies and extreme value analysis.

The Anderson-Darling estimator of the unknown parameter ξ of the DUS Lindley distribution is obtained by minimizing the.

$AD_{Z}\left(\xi |\underline{z}\right)=-n-\frac{1}{n}\sum_{r=1}^{n}\left(2r-1\right)\log\left(G_{Z_{\left(r\right)}}\left(z\right)\left(1-G_{Z_{\left(n-r+1\right)}}\left(z\right)\right)\right)$ with respect to the parameter ξ. Where $G_{Z_{\left(r\right)}}\left(z\right)$ is the CDF of rth ordered statistics of DUS Lindley distribution and is given by

GZ(r)(z)=∑j=rn(Crn)((1e−1)(e(1−(1+ξz1+ξ)e−ξz)−1))j(1−(1e−1)(e(1−(1+ξz1+ξ)e−ξz)−1))n−j

## 10 Likelihood ratio test

The likelihood ratio test (LRT) serves as a valuable tool in hypothesis testing, providing a means to assess whether a newly explored model offers a significant improvement in explaining observed data compared to the base model. Suppose *z*_*1*_*, z*_*2*_*, z*_*3*_*, …, z*_*n*_ be a random sample drawn from the DUS Lindley distribution. To test the hypothesis.

$H_{0}:g\left(z\right)=f\left(z\;;\;\xi \;\right)$ against $H_{1}:g\left(z\right)=g\left(z\;;\;\xi \right)$

The following test statistics are used to test whether the given sample has been drawn from the Lindley distribution or the DUS Lindley distribution.

$$l=\frac{L_{1}}{L_{0}}=\prod_{s=1}^{n}\frac{g\left(z_{s};\;\xi \right)}{f\left(z_{s};\;\xi \right)}$$

$$l=\prod_{s=1}^{n}\frac{\left(\frac{1}{e-1}\right)\left(\frac{\xi^{2}}{1+\xi }\right)\;\left(1+z_{s}\right)\;e^{-\xi \;z_{s}}\;\left(e^{\left(1-\left(1+\frac{\xi \;z_{s}}{1+\xi }\right)\;e^{-\xi \;z_{s}}\right)}\right)}{\left(\frac{\xi^{2}}{1+\xi }\right)\;\left(1+z_{s}\right)\;e^{-\xi \;z_{s}}}$$

After simplification, we get”

(17) $$l={\left(\frac{1}{e-1}\;\right)}^{n}\left(e^{\sum_{s=1}^{n}\left(1-\left(1+\frac{\xi \;z_{s}}{1+\xi }\right)\;e^{-\xi \;z_{s}}\right)}\right)$$

The null hypothesis can be rejected if the calculated value of test statistics in equation (17) is more significant than *D∗*, that is

$$l={\left(\frac{1}{e-1}\;\right)}^{n}\left(e^{\sum_{s=1}^{n}\left(1-\left(1+\frac{\xi \;z_{s}}{1+\xi }\right)\;e^{-\xi \;z_{s}}\right)}\right)>D^{*}$$

$$\left(e^{\sum_{s=1}^{n}\left(1-\left(1+\frac{\xi \;z_{s}}{1+\xi }\right)\;e^{-\xi \;z_{s}}\right)}\right)>D^{*}{\left(e\_1\right)}^{n}$$

$$\sum_{s=1}^{n}\left(1-\left(1+\frac{\xi \;z_{s}}{1+\xi }\right)\;e^{-\xi \;z_{s}}\right)>\log\left({D^{*}\left(e_{1}\right)}^{n}\right)$$

$$\sum_{s=1}^{n}\left(1+\frac{\xi \;z_{s}}{1+\xi }\right)\;e^{-\xi \;z_{s}}<\;n-\log\left({D^{*}\left(e_{1}\right)}^{n}\right)$$

$$\sum_{s=1}^{n}\left(1+\frac{\xi \;z_{s}}{1+\xi }\right)\;e^{-\xi \;z_{s}}<\;C^{*}$$

Such that $P\left(l>D^{*}\right)=\alpha \text{new}$.

Where $C*=n-\log\left({D^{*}\left(e_{1}\right)}^{n}\right)$ and *α* is the level of significance at which we have to test the given null hypothesis against the corresponding alternative hypothesis.

## 11 Simulation

Simulation offers a methodology for gaining insights into the dynamics of maximum likelihood estimators across varying sample sizes. This serves as a valuable compass for informed decision-making, risk mitigation, and enhancement of the dependability and efficiency of statistical analysis. By employing simulations, we gain the foresight to predict the performance of maximum likelihood estimators across a spectrum of sample sizes (30, 50, 100, 150, 250, 400, 600 and 700), including those that may be challenging to obtain in practical scenarios. In simulations involving probability distributions, we can technically choose any parameter value, but the chosen values should be within a plausible range that aligns with the distribution's characteristics and the problem's context. Employing the inverse cumulative distribution function technique within the R-software, the process iteratively executed 1000 times, enabling a comprehensive analysis of mean squared error (MSE), average bias and average variance. As presented in Table 1, the findings reveal a consistent diminishing trend in average bias, average variance, and MSE across diverse parameter values and sample sizes in the DUS Lindley distribution. This compelling evidence emphasizes that larger sample sizes consistently enhance the performance of MLEs within the realm of the DUS Lindley distribution. We used The average of the mean values (AE), average bias (AB), and average variance (AV) as a simulation measures.

**Table 1:** Estimation of average bias, average variance and MSE.

N	ξ = 0.6459			ξ = 1.1188		
	AB	AV	MSE	AB	AV	MSE
**30**	0.065703	0.005305	0.009622	0.101972	0.034109	0.044507
**50**	0.034107	0.004851	0.006015	0.089908	0.013068	0.021151
**100**	0.003768	0.001887	0.001902	0.054191	0.007269	0.010206
**150**	0.002819	0.001613	0.001621	0.020037	0.002064	0.002465
**250**	0.000946	0.000530	0.000531	0.010057	0.001672	0.001773
**400**	0.000687	0.000479	0.000479	0.006218	0.001464	0.001503
**600**	−0.002979	0.000439	0.000447	−0.004684	0.000540	0.000562
**700**	−0.000263	0.000405	0.000405	−0.006184	0.000499	0.000538
**n**	ξ = 2			ξ = 3		
	**AB**	**AV**	**MSE**	**AB**	**AV**	**MSE**
**30**	0.109718	0.049478	0.061516	0.139050	0.213503	0.232838
**50**	0.061689	0.022441	0.026247	0.109446	0.055461	0.067440
**100**	0.038907	0.019293	0.020807	0.086063	0.025735	0.033142
**150**	0.029238	0.010865	0.011719	0.068875	0.023445	0.028189
**250**	0.017030	0.006951	0.007241	0.017178	0.012904	0.013199
**400**	−0.018110	0.003604	0.003932	0.005802	0.007192	0.007226
**600**	−0.037736	0.001948	0.003372	−0.025719	0.004956	0.005617
**700**	−0.002347	0.002610	0.002615	−0.043050	0.005287	0.007140
**n**	ξ = 4			ξ = 5		
	**AB**	**AV**	**MSE**	**AB**	**AV**	**MSE**
**30**	0.343424	0.444701	0.562641	0.331790	0.793306	0.903390
**50**	0.232157	0.160469	0.214365	0.320336	0.734219	0.836834
**100**	0.203505	0.082280	0.123694	0.215907	0.488871	0.535486
**150**	0.174908	0.069214	0.099807	0.155156	0.335186	0.359259
**250**	0.099782	0.031830	0.041786	0.080740	0.151656	0.158175
**400**	0.025979	0.006447	0.007122	0.065781	0.072790	0.077118
**600**	−0.025311	0.004616	0.005257	0.081831	0.032855	0.039551
**700**	−0.039148	0.008610	0.010143	0.041836	0.020535	0.022285
**n**	ξ = 6			ξ = 9		
	**AB**	**AV**	**MSE**	**AB**	**AV**	**MSE**
**30**	0.357519	1.222594	1.350414	0.532661	1.545908	1.829636
**50**	0.307927	0.985229	1.080048	0.397215	1.289271	1.447051
**100**	0.205659	0.325669	0.367965	0.206517	0.579750	0.622400
**150**	0.170205	0.124098	0.153067	0.107127	0.355512	0.366988
**250**	0.089654	0.091838	0.099876	0.079526	0.194895	0.201220
**400**	0.038643	0.041904	0.043397	0.028938	0.096751	0.097588
**600**	−0.058877	0.030366	0.033832	0.002055	0.041568	0.041573
**700**	−0.120955	0.014296	0.028926	−0.032999	0.040590	0.041679
**n**	ξ = 12			ξ = 16		
	**AB**	**AV**	**MSE**	**AB**	**AV**	**MSE**
**30**	0.922909	2.673732	3.525492	1.358479	3.744439	5.589904
**50**	0.697756	2.609794	3.096657	1.033469	2.761239	3.829298
**100**	0.510929	1.241376	1.502424	0.803973	1.975521	2.621894
**150**	0.356419	0.782257	0.909291	0.619455	1.384804	1.768529
**250**	0.194015	0.560550	0.598192	0.399562	0.540600	0.700250
**400**	0.054803	0.178192	0.181195	0.174032	0.224438	0.254725
**600**	−0.016950	0.102794	0.103082	0.034491	0.105297	0.106487
**700**	−0.006647	0.047186	0.047230	−0.209935	0.033562	0.077635
**n**	ξ = 40			ξ = 45		
	**AB**	**AV**	**MSE**	**AB**	**AV**	**MSE**
**30**	3.120467	31.96528	41.70259	3.990677	32.37161	48.29711
**50**	2.441014	11.14246	17.10101	2.773191	12.042235	19.732823
**100**	1.561133	9.250545	11.68768	1.646063	9.7957702	12.505294
**150**	1.342766	6.134707	7.937728	0.962948	8.128833	9.056103
**250**	1.055134	4.454608	5.567915	0.481012	5.470338	5.701710
**400**	0.462429	1.800892	2.014732	0.154674	2.108304	2.132228
**600**	0.313255	1.493712	1.591840	0.053931	1.128625	1.131534
**700**	0.207784	0.950715	0.993889	0.012565	0.872771	0.872928
**n**	ξ = 7			ξ = 10		
	**AB**	**AV**	**MSE**	**AB**	**AV**	**MSE**
**30**	0.805421	1.702889	2.351591	0.909386	2.676348	3.503332
**50**	0.675816	1.141494	1.598221	0.846362	1.434491	2.150820
**100**	0.518544	0.772500	1.041388	0.513515	1.196474	1.460172
**150**	0.295340	0.489032	0.576257	0.416194	0.894722	1.067940
**250**	0.155738	0.278910	0.303165	0.251724	0.521827	0.585192
**400**	0.068045	0.130929	0.135559	0.085356	0.318695	0.325980
**600**	0.035410	0.033351	0.034605	0.017111	0.092486	0.109597
**700**	−0.017538	0.051608	0.051916	−0.195387	0.058537	0.096713
**n**	ξ = 14			ξ = 18		
	**AB**	**AV**	**MSE**	**AB**	**AV**	**MSE**
**30**	1.035011	5.835616	6.906864	1.356951	4.647477	6.488793
**50**	0.935891	3.693261	4.569152	1.126673	3.940466	5.209858
**100**	0.725905	1.948971	2.475909	1.016758	3.093248	4.127045
**150**	0.574382	1.687493	2.017407	0.857790	2.267316	3.003119
**250**	0.314968	0.996542	1.095748	0.456192	1.690821	1.898933
**400**	0.158119	0.438796	0.463798	0.161546	0.817502	0.843599
**600**	0.043948	0.207934	0.209866	0.068875	0.283312	0.288056
**700**	0.036051	0.171864	0.173165	0.044573	0.147806	0.149793
**n**	ξ = 20			ξ = 25		
	**AB**	**AV**	**MSE**	**AB**	**AV**	**MSE**
**30**	1.736256	7.080908	10.09549	2.095845	15.52148	19.91404
**50**	1.327615	6.372609	8.135171	1.6827561	7.967285	10.798953
**100**	1.143962	5.503591	7.812240	1.392518	6.093752	8.032858
**150**	0.901639	4.390682	5.203634	1.019624	5.148736	6.188360
**250**	0.658317	2.701547	3.134928	0.587931	3.374953	3.720616
**400**	0.358141	1.558064	1.686329	0.231876	1.537856	1.5916224
**600**	0.097324	0.478256	0.487728	0.053948	0.6210837	0.6239940
**700**	0.031673	0.293312	0.294315	0.023834	0.434877	0.435445
**n**	**ξ=**35			**ξ=**50		
	**AB**	**AV**	**MSE**	**AB**	**AV**	**MSE**
**30**	2.782639	24.12592	31.869001	4.314619	55.97861	74.59455
**50**	2.234012	9.672583	14.663393	3.732704	12.716885	26.649964
**100**	1.946453	8.192645	11.981324	3.149763	10.259246	20.180253
**150**	1.247938	6.284691	7.842040	2.235848	8.177419	13.176435
**250**	0.651029	4.0278549	4.451694	1.429548	5.624308	7.6679155
**400**	0.301245	2.067362	2.1581105	0.541759	2.821973	3.1154758
**600**	00.092537	0.960835	0.969398	0.130947	0.756927	0.7740741
**700**	0.019283	0.594631	0.595003	0.035715	0.501946	0.503221

${\bar{X}}_{r}$

For this R-software has been employed to calculate values. Fig. 10, Fig. 11, Fig. 12, Fig. 13, Fig. 14, Fig. 15, Fig. 16, Fig. 17, Fig. 18, Fig. 19, Fig. 20, Fig. 21, Fig. 22, Fig. 23, Fig. 24, Fig. 25 illustrates the simulation histograms showing the diverse outcomes across various sample sizes and parameters.

**Fig. 10:**
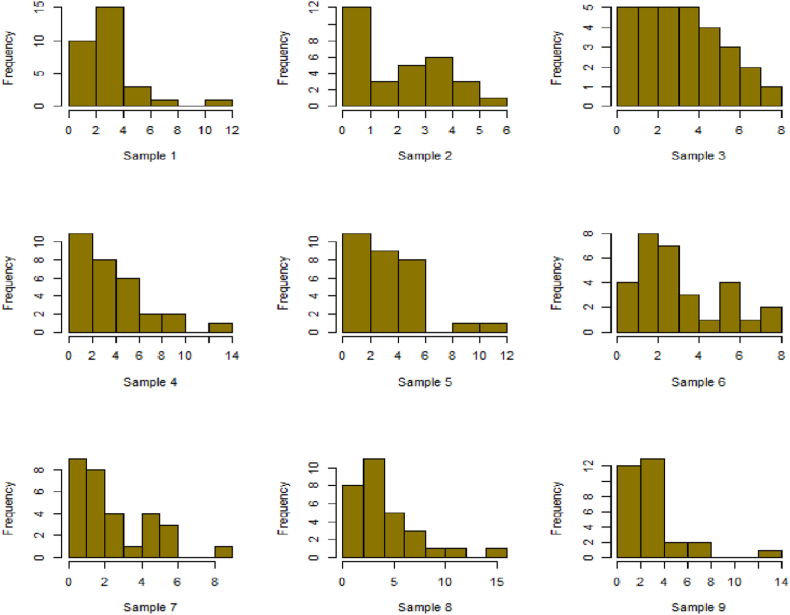
Simulation histogram for ξ = 0.6459, n = 30.

**Fig. 11:**
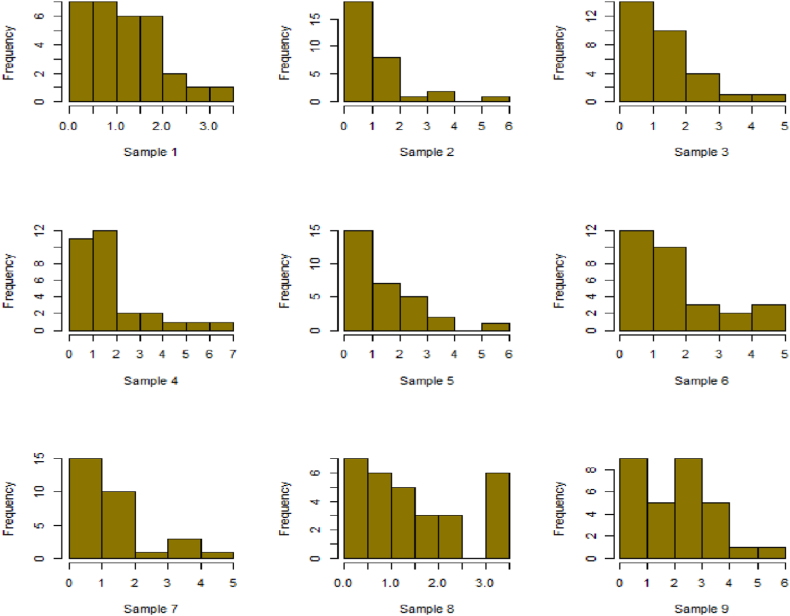
Simulation histogram for ξ = 1.1188, n = 30.

**Fig. 12:**
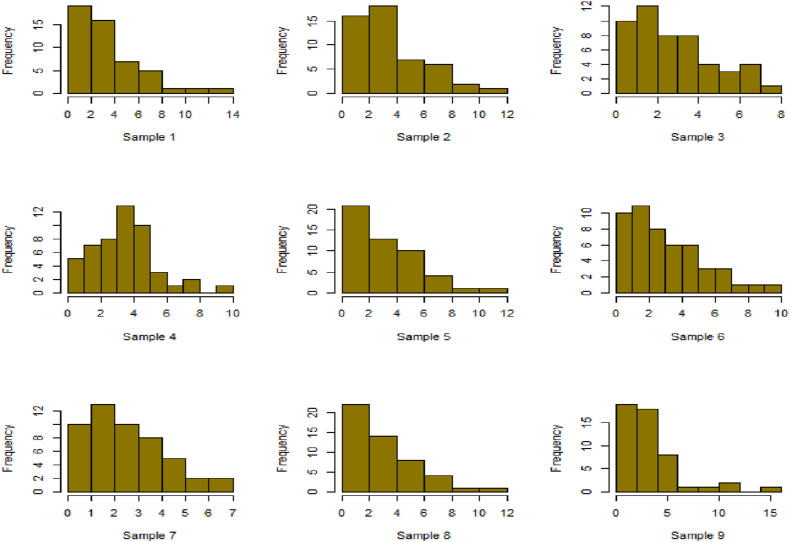
Simulation histogram for ξ = 0.6459, n = 50.

**Fig. 13:**
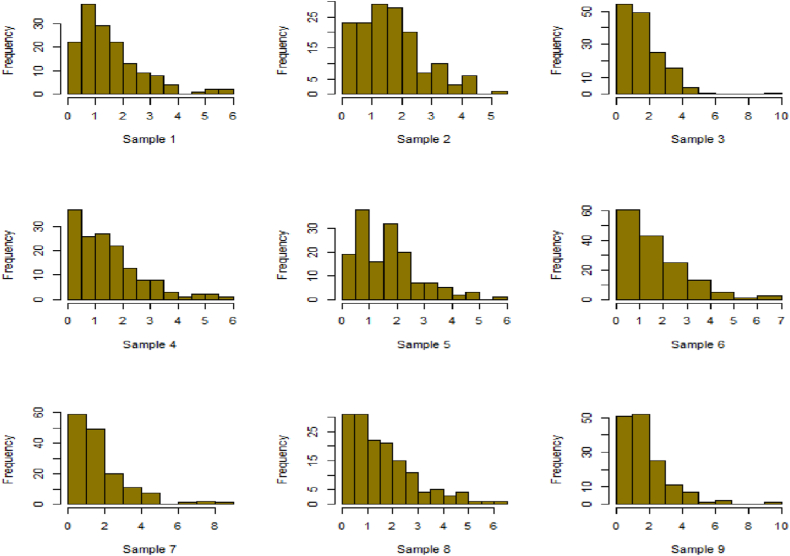
Simulation histogram for ξ = 1.1188, n = 100.

**Fig. 14:**
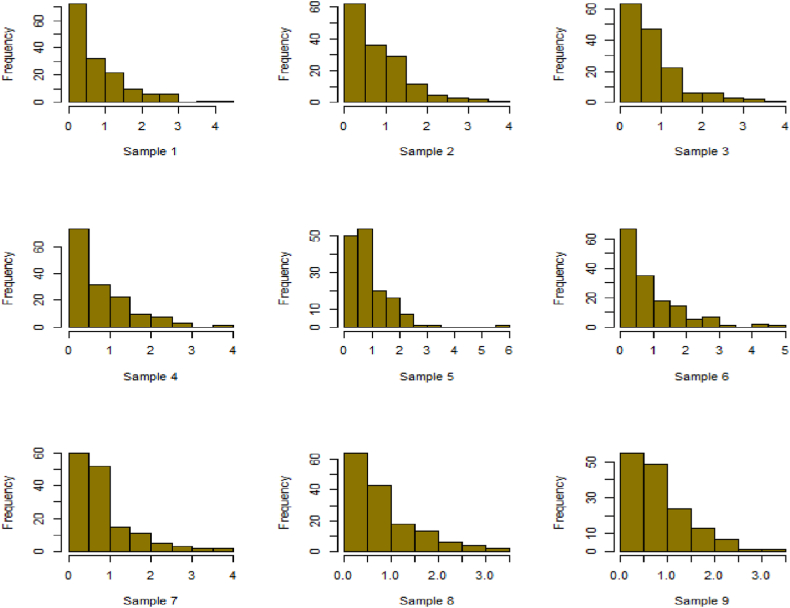
Simulation histogram for ξ = 2, n = 150.

**Fig. 15:**
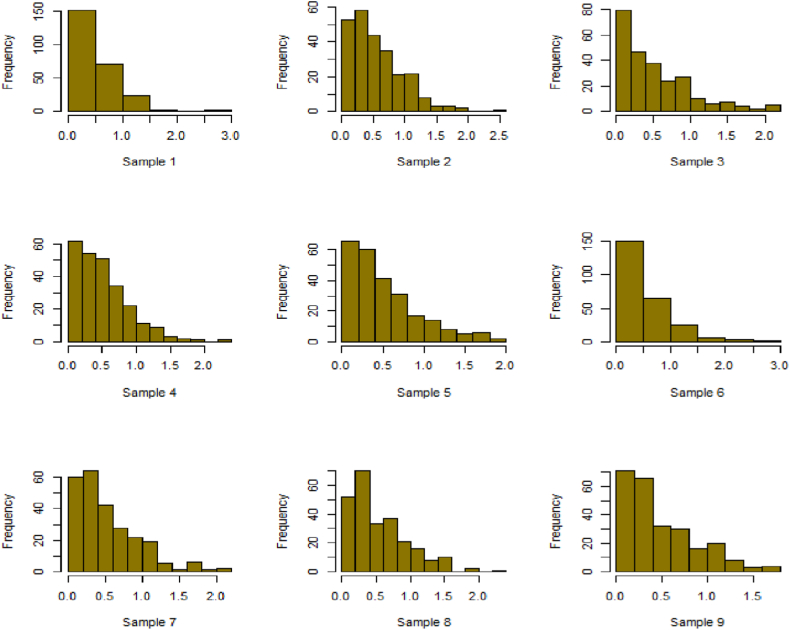
Simulation histogram for ξ = 3, n = 250.

**Fig. 16:**
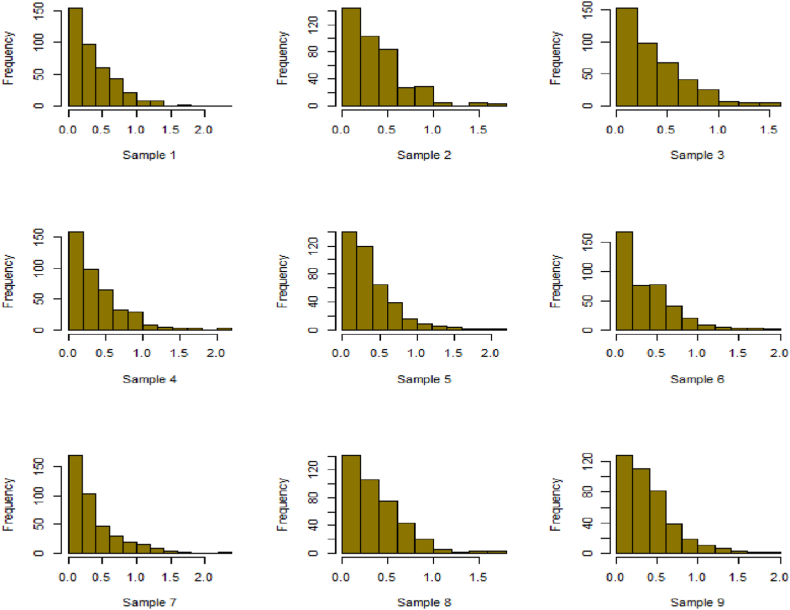
Simulation histogram for ξ = 4, n = 400.

**Fig. 17:**
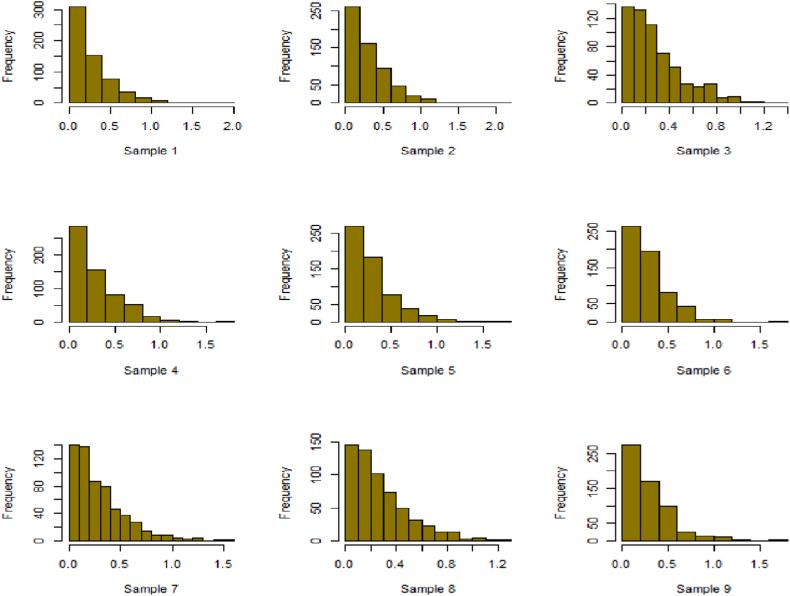
Simulation histogram for ξ = 5, n = 600.

**Fig. 18:**
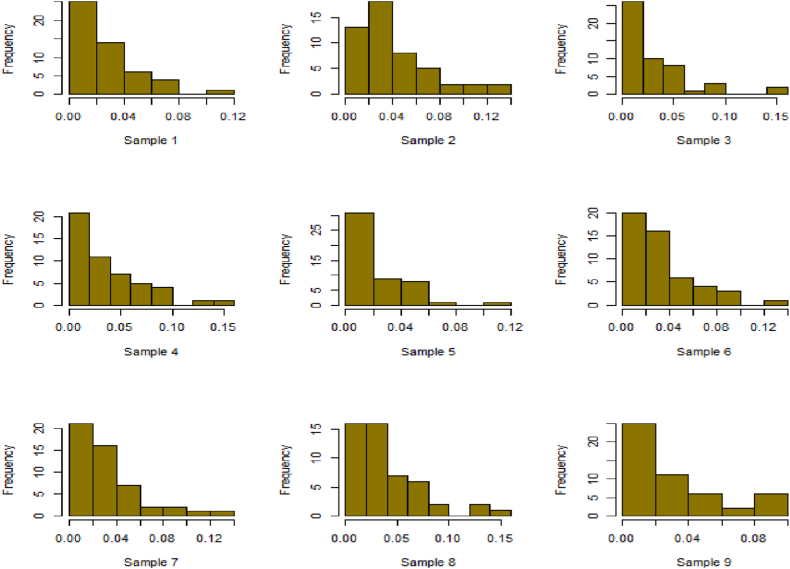
Simulation histogram for ξ = 40, n = 50.

**Fig. 19:**
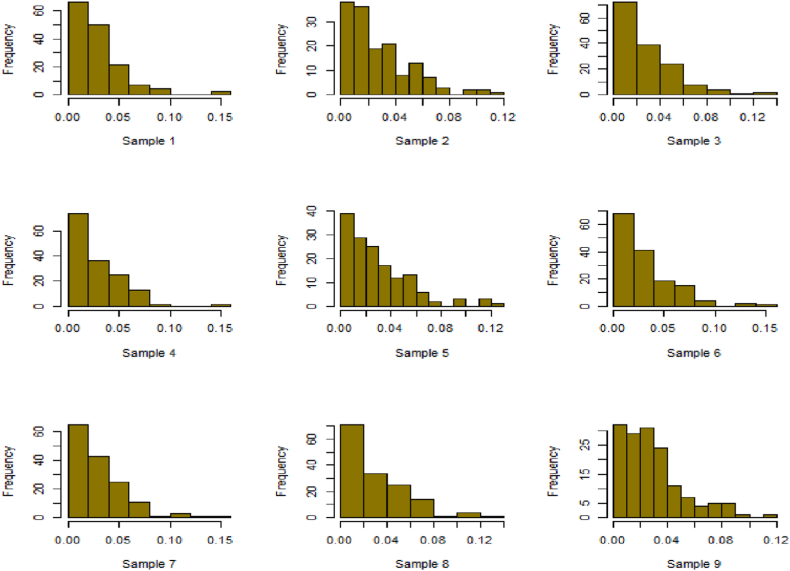
Simulation histogram for ξ = 45, n = 100.

**Fig. 20:**
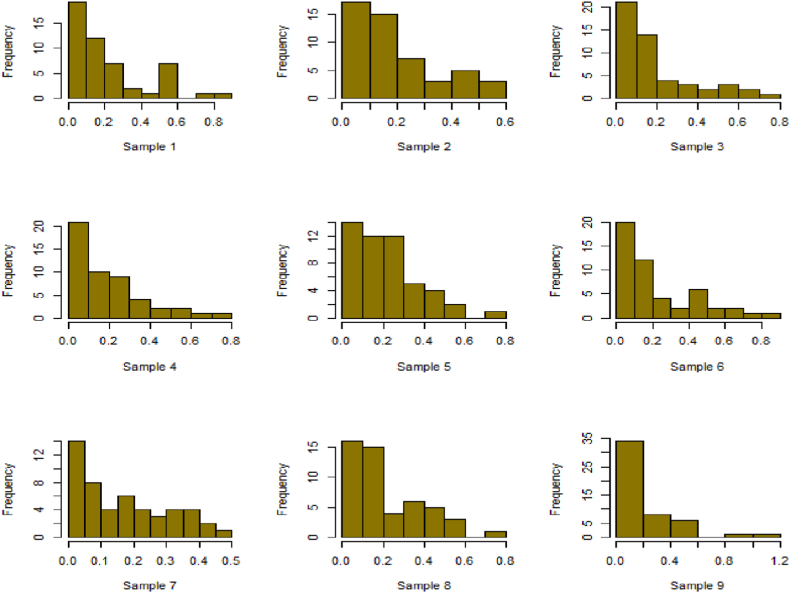
Simulation histogram for ξ = 7, n = 50.

**Fig. 21:**
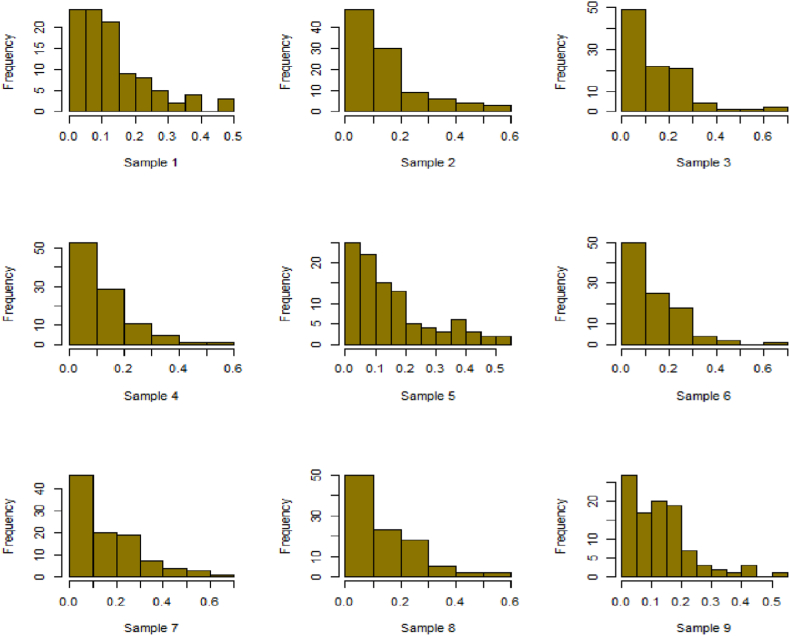
Simulation histogram for ξ = 10, n = 100.

**Fig. 22:**
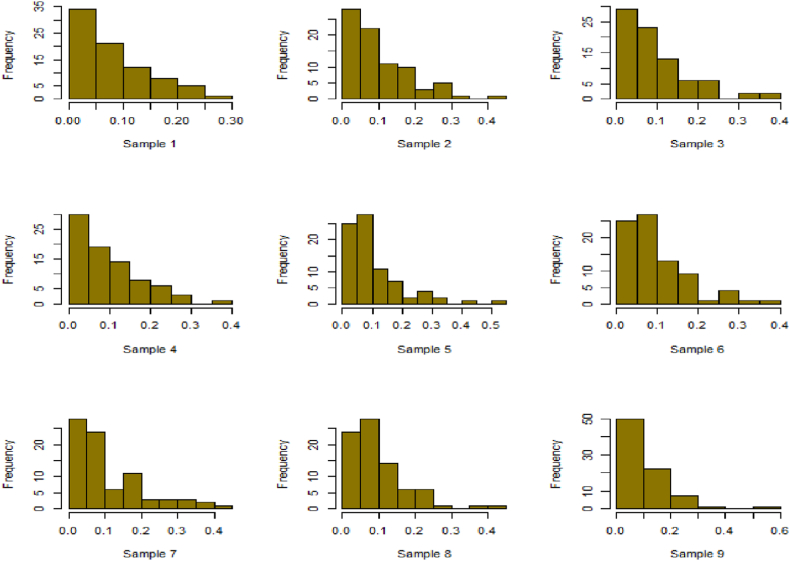
Simulation histogram for ξ = 14, n = 250.

**Fig. 23:**
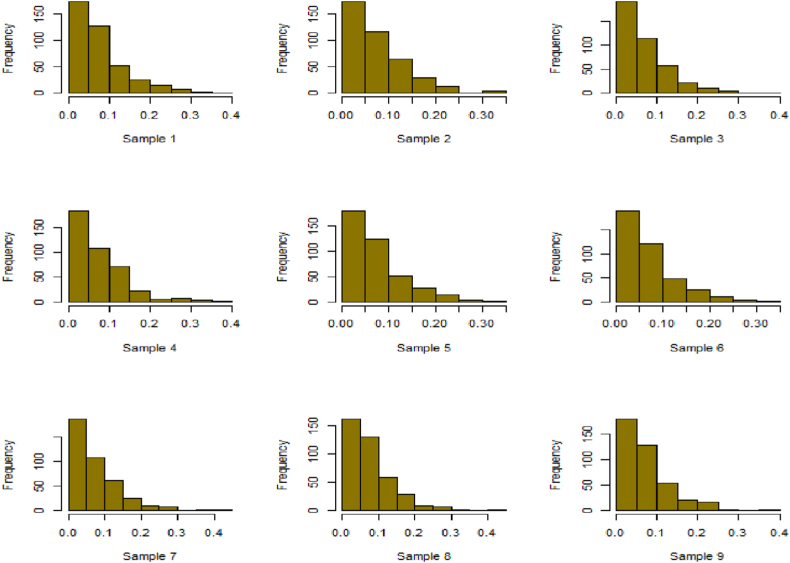
Simulation histogram for ξ = 18, n = 400.

**Fig. 24:**
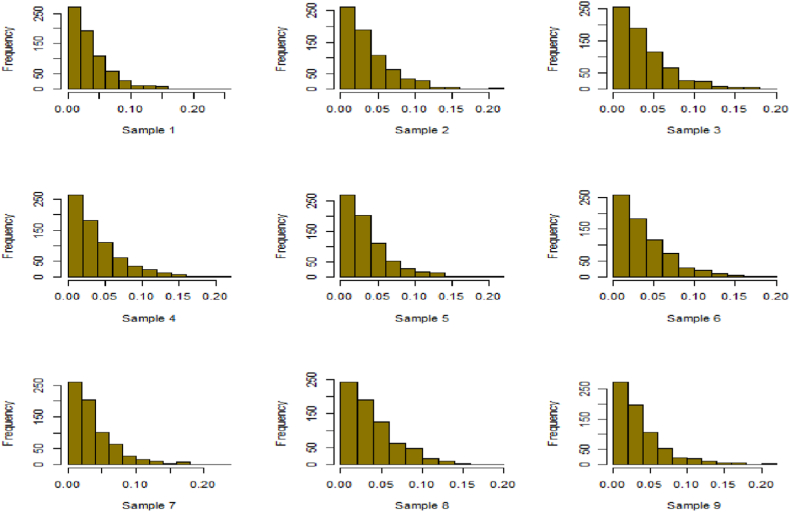
Simulation histogram for ξ = 35, n = 700.

**Fig. 25:**
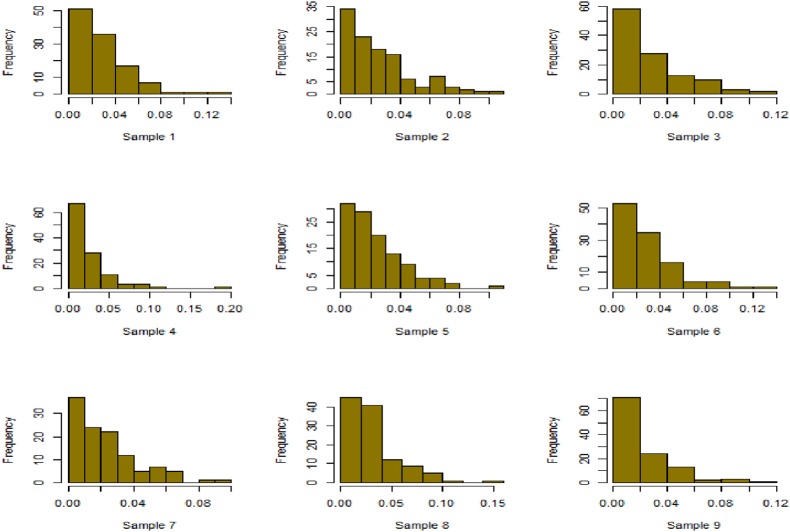
Simulation histogram for ξ = 50, n = 700.

## 12 Applications

This section evaluates the effectiveness of the newly proposed DUS Lindley distribution using two real-world datasets. Lindley, exponential, and DUS exponential distributions are also applied to the same datasets to provide a comprehensive comparison. Estimating unknown parameters is conducted using R software, ensuring robust and accurate parameter estimates.

**Data set 1:** The following data set of observations studied by Hinkley [42] is related to successive precipitation of thirty days of March (in inches).

0.77,1.74, 0.81, 1.20, 1.95, 1.20, 0.47, 1.43, 3.37, 2.20, 3.00, 3.09, 1.51, 2.10, 0.52, 1.62, 1.31, 0.32, 0.59, 0.81, 2.81, 1.87, 1.18, 1.35, 4.75, 2.48, 0.96, 1.89, 0.90, 2.05.

**Data set 2:** This data set by Abouammah et al. (2000) [43] represents the blood cancer (leukaemia) of 40 patients in Saudi Arabia of Ministry of Health hospitals. The data is mentioned below.

0.315 0.496 0.616 1.145 1.208 1.263 1.414 2.025 2.036 2.162 2.211 2.37 2.532 2.693 2.805 2.91 2.912 3.192 3.263 3.348 3.348 3.427 3.499 3.534 3.767 3.751 3.858 3.986 4.049 4.244 4.323 4.381 4.392 4.397 4.647 4.753 4.929 4.973 5.074 5.381.

The descriptive statistics of data sets 1 and 2, respectively are summarised in Table 2, Table 3. Table 4 outlines the maximum likelihood estimate (MLE) and standard error for the distributions parameter based on data sets 1 and 2. To assess model performance, we apply several evaluation criteria: The Bayesian Information Criterion (BIC), Hannan-Quinn Information Criterion (HQIC), Corrected Akaike Information Criterion (AICC), Akaike Information Criterion (AIC) and log-likelihood (-2logL). A summary of these criteria is presented in Table 5, which provides an overview of the model performance. Lower values of AIC, AICC, HQIC and BIC indicate a better fit for the respective distribution. Additionally, Table 5 includes the results of the Kolmogorov-Smirnov test and the corresponding p-values for each distribution, offering further insight into the model's fit.

**Table 2:** The descriptive statistics of data set 1.

Min	Q_1_	Median	Mean	Q_3_	Max	Skewness	Kurtosis
0.320	0.915	1.470	1.675	2.087	4.750	1.086682	4.206884

**Table 3:** The descriptive statistics of data set 2.

Min	Q_1_	Median	Mean	Q_3_	Max	Skewness	Kurtosis
0.315	2.199	3.348	3.141	4.264	5.381	−0.416727	2.273833

**Table 4:** Estimates of distribution parameter.

Data Set	Distribution	Parameter	MLE	Standard error
1	DUS Lindley	ξ	1.1188	0.1360
	Lindley	ξ	0.9097	0.1247
	DUS Exponential	ξ	0.7799	0.1217
	Exponential	ξ	0.5970	0.1090
2	DUS Lindley	ξ	0.6459	0.0649
	Lindley	ξ	0.5269	0.0607
	DUS Exponential	ξ	0.4173	0.0556
	Exponential	ξ	0.3184	0.0503

**Table 5:** Comparison of model criterion values.

Data Set	Distribution	-2logL	AIC	AICC	HQIC	BIC	K-S Test	p-value
1	DUS Lindley	82.9530	84.9530	85.0959	85.4013	86.3542	0.1578	0.4436
	Lindley	86.2875	88.2875	88.4303	88.7357	89.6886	0.1882	0.2383
	DUS Exponential	86.2031	88.2031	88.3459	88.6513	89.6043	0.1988	0.1868
	Exponential	90.9488	92.9488	93.0916	93.39705	94.3450	0.2352	0.0724
2	DUS Lindley	154.5713	156.5713	156.6766	157.1819	158.2602	0.2168	0.0515
	Lindley	160.5012	162.5012	162.6065	163.1118	164.19	0.2406	0.0195
	DUS Exponential	163.5171	165.5171	165.6224	166.1277	167.206	0.2726	0.0052
	Exponential	171.5563	173.5563	173.6616	174.1669	175.2452	0.3002	0.0015

It can be observed from Table 5, that the DUS Lindley distribution yields lower values for -2logL, AIC, AICC, HQIC, and BIC compared to the Lindley, DUS exponential, and exponential distributions. This strongly indicates that the DUS Lindley distribution is the most effective model for accurately representing the given data sets. Moreover, the Kolmogorov-Smirnov test results and the corresponding p-values further support the conclusion that the DUS Lindley distribution offers a superior fit for the data.

**Fig. 26:**
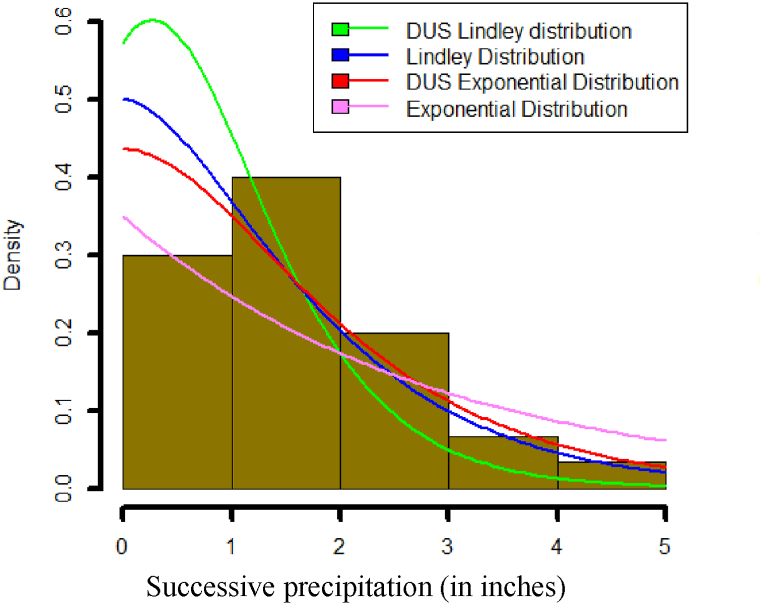
Fitted density curves of data set 1.

**Fig. 27:**
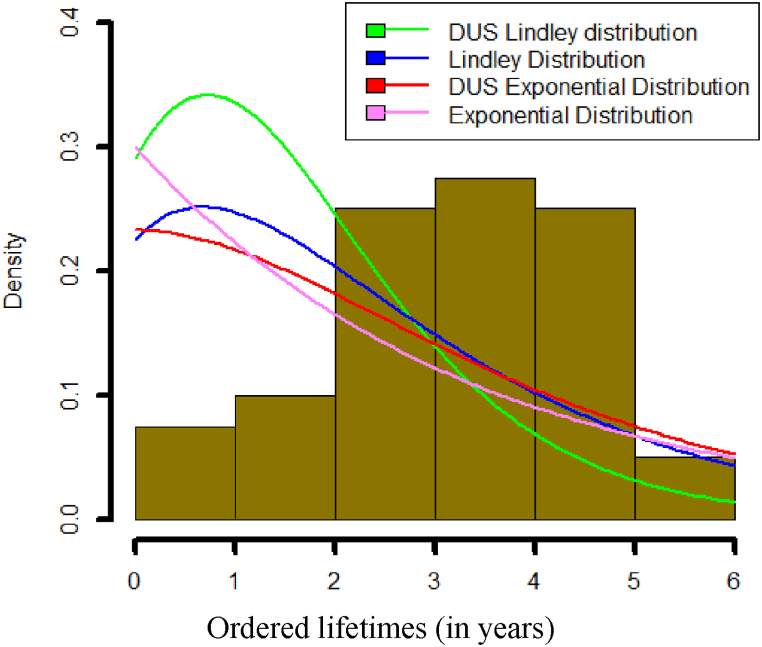
Fitted density curves of data set 2.

**Fig. 28:**
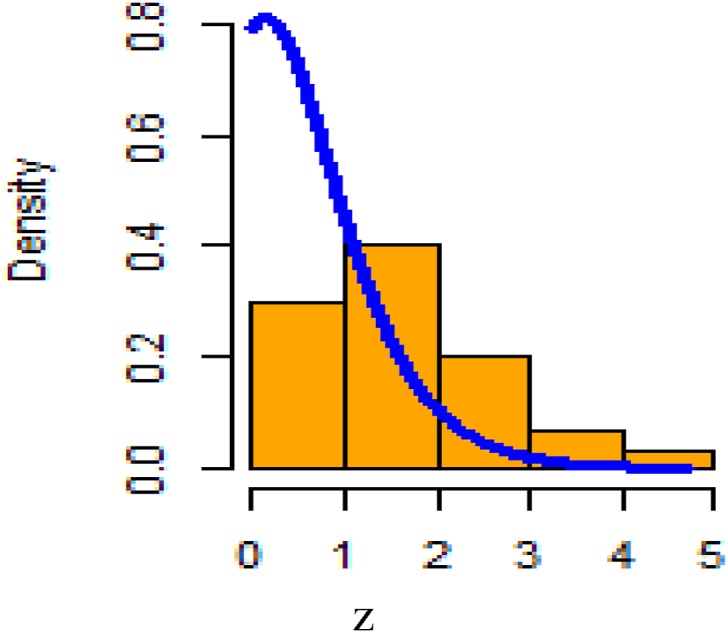
Fitted density curve and histogram of data set 1.

**Fig. 29:**
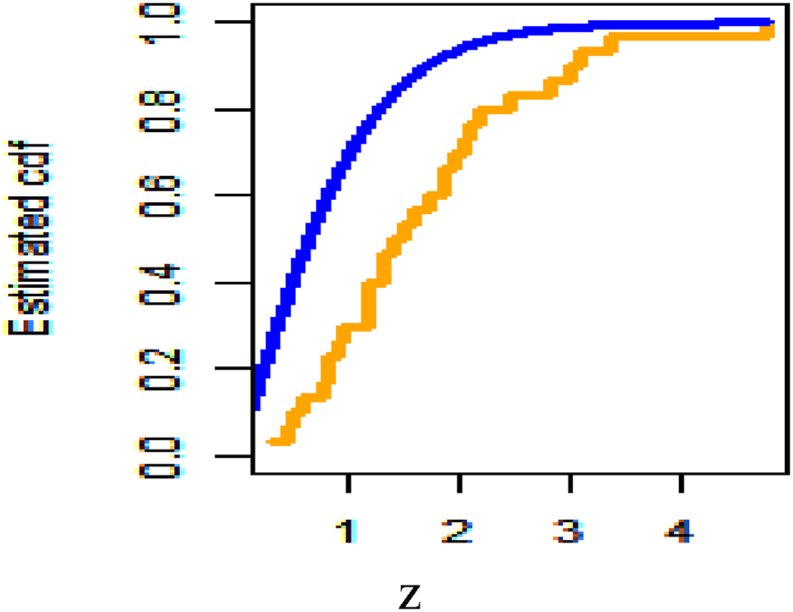
Theoretical CDF versus empirical CDF of data set 1.

**Fig. 30:**
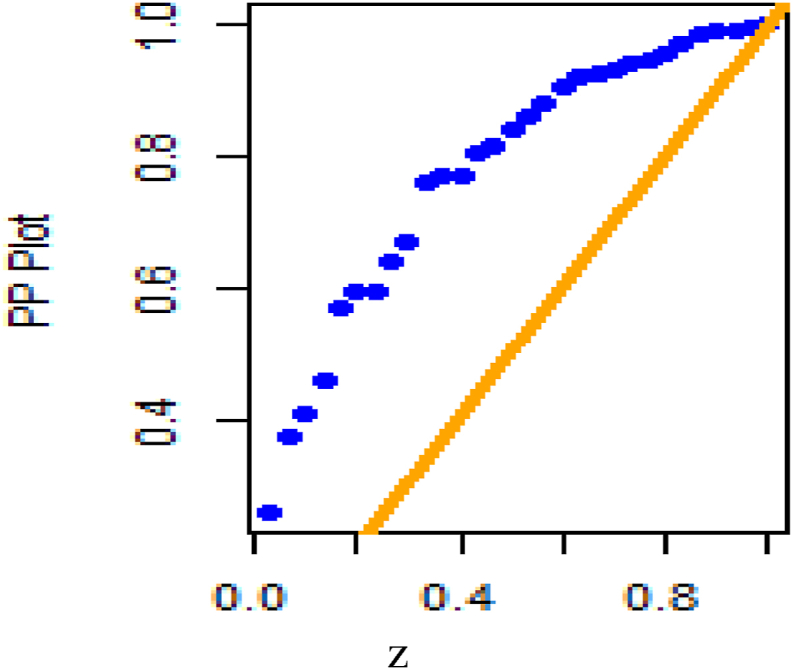
P-P Plot for DUS lindley distribution of data set 1.

**Fig. 31:**
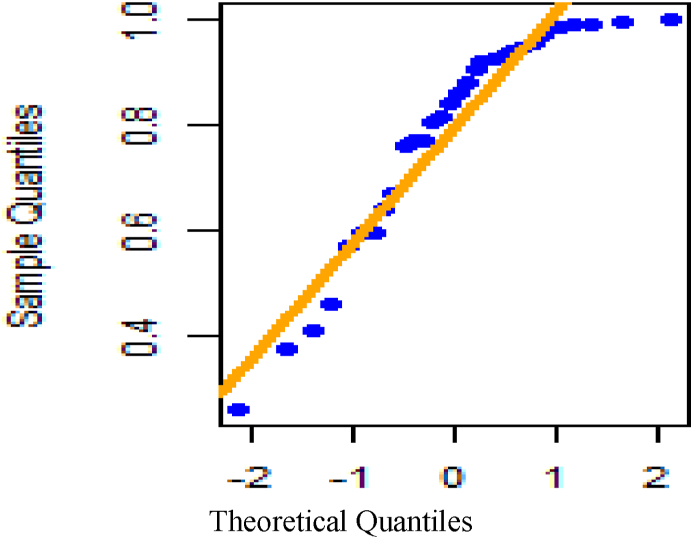
Q-Q Plot for DUS lindley distribution of data set 1.

**Fig. 32:**
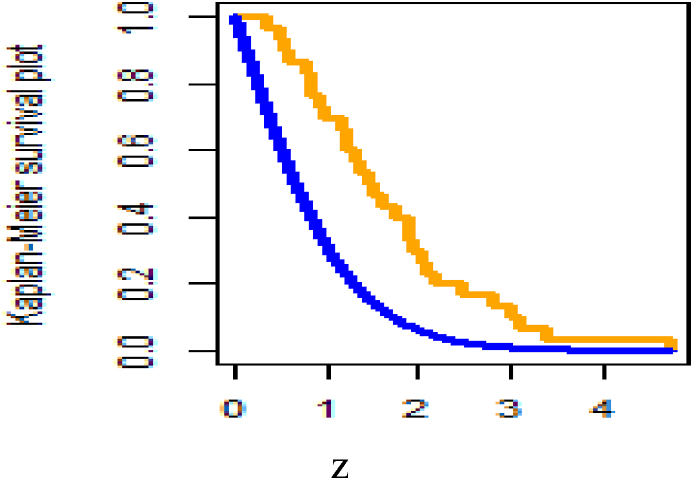
Kaplan- Meier survival plot versus empirical survival plot of data set 1.

Fig. 26 shows fitted density curves of data set 1 and Fig. 27 shows fitted density curves of data set 2, Fig. 28 shows the fitted density curve of data set 1, Fig. 29 shows theoretical CDF versus empirical CDF of data set 1, Fig. 30 shows P-P Plot for DUS Lindley Distribution of data set 1, Fig. 31 shows Q-Q Plot for DUS Lindley Distribution of data set 1, and Fig. 32 shows Kaplan- Meier survival plot versus empirical survival plot of data set 1.

## 13 Conclusion

This paper introduces the novel DUS Lindley distribution through a new transformative process known as the DUS transformation. The transformed model exhibits enhanced flexibility compared to the original distribution, allowing for a more versatile representation of the underlying data. To illustrate its characteristics, PDF plots and corresponding distribution and survival functions for select parameter values provide an idea about the distribution's behaviour. Some statistical properties of the proposed distribution are rigorously computed, including moments, conditional moments, MRL, MPL, moment generating function, characteristics function, PDF of *r*th of order statistics, Lorenz and Bonferroni curves, and entropy measures. A likelihood ratio test is employed to evaluate the model's effectiveness. The unknown model parameter is estimated using estimation methods such as the MLE, LSE, WLSE, CVMS, and ADE method.

Furthermore, applying the DUS Lindley distribution emerges as a helpful guide in evaluating system reliability, focusing on individual component lifetime distributions. In reliability section, the values like 0.9779, 0.9629, 0.9241, 0.8769, 0.8505 provide a comprehensive and insightful assessment that the overall probability of a system is functioning correctly. An extensive simulation analysis is undertaken to assess the efficiency of MLE. The model's usefulness is illustrated using two real datasets. The DUS Lindley distribution emerges as a robust fit, outperforming other established distributions such as Lindley, exponential and DUS exponential. This finding highlights the DUS Lindley distribution as a superior alternative in real-world data analysis, presenting a valuable contribution to the existing distribution landscape.

Future research directions could explore the application of the DUS Lindley distribution in diverse fields, such as survival analysis and financial modeling, to assess its robustness across various data types. Additionally, extending the DUS transformation to other baseline distributions could yield new models with enhanced flexibility. Comparative studies involving more complex real-world data sets could further validate the distribution's effectiveness and uncover potential areas for refinement. Focus on adapting this distribution to more complex datasets and expanding its use in different domains makes it as a valuable tool for future statistical modeling endeavors.

## Data availability statement

Data included in article/supplementary material is referenced in the article.

## CRediT authorship contribution statement

**Danish Qayoom:** Writing – review & editing, Writing – original draft, Formal analysis, Data curation, Conceptualization. **Aafaq A. Rather:** Writing – original draft, Methodology, Investigation, Data curation, Conceptualization. **Najwan Alsadat:** Visualization, Supervision, Software, Resources, Investigation. **Eslam Hussam:** Validation, Software, Resources, Methodology. **Ahmed M. Gemeay:** Writing – original draft, Supervision, Software, Resources, Project administration, Methodology, Formal analysis.

## Declaration of competing interest

The authors declare that they have no known competing financial interests or personal relationships that could have appeared to influence the work reported in this paper.
